# Mechanistic Insights into Emulsion Destabilization by Electric Fields

**DOI:** 10.1021/acs.langmuir.5c02307

**Published:** 2025-09-22

**Authors:** Alexandra Alicke, Nick O. Jaensson, Jan Vermant

**Affiliations:** † Department of Materials, 111950 ETH Zurich, Vladimir-Prelog-Weg 5, 8093 Zurich, Switzerland; ‡ Department of Mechanical Engineering, 685177 Eindhoven University of Technology, P.O. Box 513, 5600 MB Eindhoven, The Netherlands

## Abstract

Although stable emulsions are often desirable, in some applications, they must be destabilized. One common approach is to apply electric fields to promote droplet coalescence, yet the underlying mechanisms remain poorly understood due to the sometimes subtle interplay of hydrodynamics, capillarity, intermolecular forces, and both interfacial and Maxwell stresses. Here, we use a modified dynamic thin film balance technique to simulate electrocoalescence in two types of systems, namely “surface active” and “rheologically active” interfaces. Despite their distinct stabilization mechanisms, we find that the key factor influencing electrocoalescence is the same in all cases: the local film thickness, which directly affects the magnitude of the Maxwell pressure. For nonionic surfactant films, we identify two distinct regimes: (i) a hydrodynamics-dominated regime, where relatively small electric pressures (∼Pa range) are sufficient to break the film, and (ii) a regime in which intermolecular forces stabilize the Newton Black Film, increasing the required breakup pressure to the ∼ kPa range. Asphaltene-laden films, in the limit of insoluble interfaces, form a representative example of rheologically complex interfaces. We find that elastic properties stabilize these films at significantly larger thicknesses, rendering electric fields ineffective—unless demulsifiers are introduced to promote local heterogeneity and thinning. This study provides new insight into how electrostatic fields destabilize emulsions and suggests new avenues for developing more efficient destabilization strategies.

## Graphical abstract

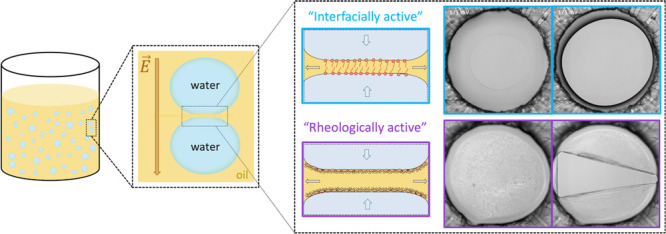

## Introduction

While emulsion stability is often desirable in fields such as food and pharmaceutical sciences, it can pose significant challenges in others. For instance, stable water-in-crude oil emulsions are problematic for transportation and separation processes. Consequently, a lot of effort has been put into efficiently ‘breaking’ these emulsions. Some strategies include the use of chemical demulsifiers and application of external electric fields to enhance droplet coalescence, thus increasing droplet sizes and improving sedimentation rates in separators. Typically, both methods are employed in tandem; however, their precise effects on droplet interactions are still not completely understood, as recently discussed in.^1–4^ Specifically, it is not clear if the electric fields merely deform the droplets or also influence the interfacial layers, and why their effectiveness is sometimes limited without chemical demulsifiers. Thinking about how environmentally unfriendly the addition of extra chemicals can be or how much energy must be used to generate the AC fields used in electrocoalescers, also the ecological impact could be significantly reduced by designing more efficient destabilizing processes.^5–7^ Beyond this particular application, a fundamental understanding of emulsion stabilization mechanisms is essential for designing effective destabilization strategies across a wide range of emulsions.

In order to do so, first the nature of emulsion stability must be well understood. Even though crude oils are complex mixtures encompassing different surface active components, the role of asphaltenes in emulsion stability has been recognized and studied extensively.^8–14^ In previous work,^15^ we clearly showed that asphaltenes impart significant mechanical strength to oil–water interfaces, acting more as “rheologically active” than as “surface active” agents, in agreement with observations from earlier works.^12,16–18^ Specifically, we observed a monotonic increase in both shear and dilatational elastic moduli with increasing surface coverage, an effect far more pronounced than changes in surface pressure or interfacial tension, suggesting that densely populated interfaces become increasingly difficult to destabilize due to their elastoplastic rheological response. To achieve coalescence, two droplets must come into close contact, allowing the intervening liquid film to drain until it ruptures, thereby merging the droplets. In thin film drainage flow, there is an intricate coupling between capillarity, intermolecular and hydrodynamic stresses,^19^ with the mechanical properties of the two approaching interfaces introducing another degree of complexity.

Chemical demulsifiers are designed to competitively adsorb at the droplet’s surface, displacing the indigenous surface active components. They typically contain both hydrophilic and hydrophobic moieties to ensure surface activity, along with high diffusivity in the oil phase for rapid migration to the interface.^20^ Common chemistries include resin alkoxylates, polyesters, or high-molecular-weight block copolymers,^3,21^ and recent work focused on so-called “green demulsifiers” to decrease their environmental impact.^7^

Demulsifier efficiency has been studied empirically at the emulsion scale and through adsorption studies, mainly focusing on their ability to lower interfacial tension.^22–24^ While initially they were thought to work by replacing the asphaltenes and other crude oil surface active components from the interface, the presence at only ppm concentrations in the bulk is by far not sufficient to do so, as pointed out by Sjöblom et al.^2^ The prevailing hypothesis is that once adsorbed, demulsifiers decrease the interfacial tension, enhancing film drainage by suppressing Marangoni flows.^22,25,26^ This, however, assumes that the stability in these emulsions is governed by gradients in interfacial tension, which in turn implies that asphaltenes behave as simple surfactants. While this can be the case at very low concentrations and/or highly soluble systems where asphaltenes would be present as single molecules, for the most problematic cases of densely packed interfaces where stability is high due to the pronounced mechanical properties, more complicated phenomena must take place. Some studies suggest demulsifiers disrupt the interface’s elastic nature, as inferred from interfacial shear rheology measurements^11,27^ and thin film drainage experiments.^28^ Nonetheless, how exactly demulsifiers enhance coalescence, particularly when combined with electric fields, remains poorly understood.

Electric fields have long been employed to enhance droplet coalescence in industrial processes,^29,30^ yet the fundamental mechanisms remain partially unclear. When applied, they polarize droplets, increasing contact probability and promoting thin film drainage.^31,32^ Key parameters include field strength, droplet size, volume fraction ϕ_*w*_, and fluid properties.^33,34^ While DC fields provide constant force, at high ϕ_*w*_ there is a risk of short-circuiting due to aqueous disperse phase chain formation across the electrodes. Thus, AC fields - typically above ∼ kHz - are preferred to prevent this issue.^35^ Electrocoalescence in crude oil systems has been explored across multiple length scales. Large-scale and lab-scale bulk emulsion studies employ methods like bottle tests and NMR to track sedimentation fronts, evaluating separation efficiency, critical voltages, and optimal demulsifier dosages.^35–39^ Droplet-level experiments and microfluidic approaches measure coalescence times and frequencies, offering qualitative insights into how factors like temperature and fluid composition affect coalescence.^18,28,40–43^ However, a fundamental understanding remains elusive. Few studies have addressed thin film dynamics directly, focusing instead on qualitative observations of stability of diluted bitumen films and the effects of aging, concentration, and temperature.^44–48^ A systematic correlation between interfacial rheological properties and thin film stability is still lacking, hindering a clearer understanding of the electrocoalescence mechanisms.

Very few studies have examined the impact of electric fields on thin liquid films. Most works have focused on identifying critical rupture voltages in polymer,^49^ protein,^50^ and diluted bitumen films,^51,52^ following ideas introduced by Scheludko in the 1960s.^53^ Electric fields may act in multiple ways: macroscopically, through Maxwell stresses affecting fluid flow and droplet deformation,^54,55^ or by facilitating hole nucleation in thin films, akin to electroporation in vesicles.^56,57^ Molecular and particle-based simulations (MD and DPD) have provided some molecular-scale insights,^58^ particularly into how demulsifiers function and films break.^1,57,59–61^ Specifically for asphaltene-laden interfaces, there is the hypothesis that the electric field can directly interact with the asphaltenes given their high polarity and consequently affect interfacial stability.

Hence, the goal of this work is to understand the role of interfacial rheology in complex interfaces on electrocoalescence. We employ a dynamic thin film balance technique combined with an electric-field setup to examine how asphaltene-stabilized films drain and correlate these observations with measured interfacial rheological properties. This approach reveals the origin of film stability and demonstrates how applying electric fields, in concert with chemical demulsifiers, can facilitate destabilization. To contextualize our findings, we compare these “complex interfaces” to a “simple” nonionic surfactant system and complement the analysis with finite element method (FEM) simulations. Although the stabilizing mechanisms differ, rupture in both systems is governed by the same factor: the evolution of film thickness. Thinner regions amplify the electric field, ultimately causing film breakup. This indicates that the electric field predominantly acts on a continuum scale and rationalizes why chemical demulsifiers, by promoting local thinning, enhance coalescence under electric fields. This understanding provides new fundamental insights into how electric fields act on thin liquid films and consequently on emulsion destabilization.

## Theoretical Background

In this section, we introduce the problem under investigation, present the governing equations for the FEM simulations, and discuss the key simplifying assumptions and dimensionless parameters. Given their high salinity, brine droplets suspended in a low-permittivity crude oil phase can be approximated as perfect conductors in a perfect dielectric medium.^55,59,62^ Figure 1 illustrates the head-on approach of two such droplets, where the intervening liquid film drains due to the Laplace pressure difference. In addition, a potential Φ_0_ is applied at the top boundary, establishing the electric field **E**. Inside the conductive droplets, charges redistribute to the surface, rendering the electric field within them zero. In the far-field limit, each droplet behaves like a dipole. This dipole–dipole interaction between droplets generates an attractive force, ultimately accelerating film drainage.^32,33^

**Figure 1:**
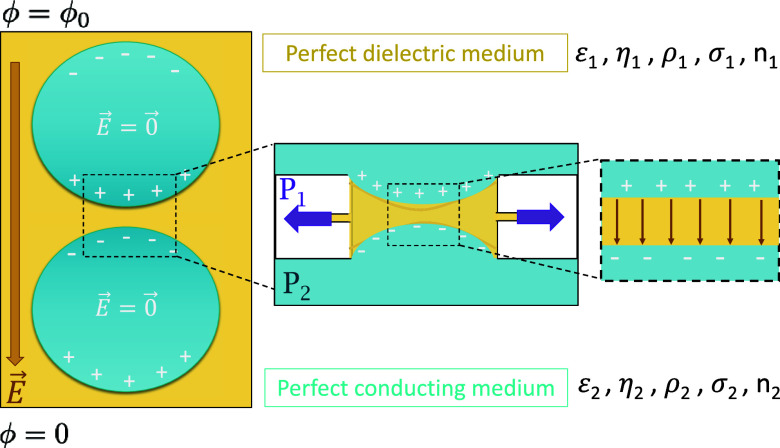
Schematics of the early stage in electrocoalescence: two conducting droplets (phase 2) approach in a perfect dielectric medium (phase 1) under an external electric field **E**. The center illustration shows the liquid film of continuous phase draining as mimicked experimentally using the electric dynamic thin film balance (DTFB) technique.

The center illustration shows the corresponding system as mimicked experimentally in a dynamic thin film balance setup (DTFB, see section Electric Thin Film Balance): a perfect dielectric film at pressure *P*_1_ surrounded by the conducting medium having a hydrostatic pressure *P*_2_. Film drainage is driven by a capillary pressure Δ*P*. Applying an electric potential across the film polarizes both sides, introducing an additional normal compressive component *P*_E_ driving drainage, which depends on film thickness and magnitude of the electric field. As will be detailed in section Experimental Procedure, different pressure contributions act to either drain or stabilize the film, which depend on the external driving forces and fluid properties. The inset depicts, as an approximation, a planar thin film forming in the center and in this case the electric field inside the film is uniform.

### Governing Equations—Electric Fields

The starting point are Maxwell’s equations for electromagnetism, and the following simplifications apply here:

(i) Effects of the electric field only (**E**) (i.e no magnetic field, **B** = **0**);
(ii) Perfect dielectric film in perfect conducting medium;

From (i), it follows that from the full set of Maxwell’s equations Gauss’ law remains:

$$\nabla \cdot E=\frac{1}{\epsilon_{0}}\rho_{c}$$ 1

where ρ_c_ is the charge density and ϵ_0_ is the permittivity of free space. From (ii), it follows that the charge relaxation time in the conducting medium is negligible, allowing the system to instantly adjust to any changes. Consequently, no free charges accumulate in the bulk, no significant motion is expected to occur, and the current density **J** effectively vanishes. Then, charge conservation yields

$$\frac{\partial \rho_{c}}{\partial t}=-\nabla \cdot J=0$$ 2

Further assuming an irrotational field (∇ × **E** = **0**), which follows from Faraday’s law, the electric field is the gradient of the potential (**E** = −∇Φ) and from Gauss’ law it follows that the divergence of the electric field is zero:

∇·E=0 3

meaning that the electric field problem reduces to an electrostatic problem. For a planar film, we can approximate the magnitude of the electric field *E* inside a planar film as

$$\left|E\right|=E\approx -\frac{\Delta \Phi }{h}$$ 4

where ΔΦ is the applied constant potential and *h* is the thickness of the film. Moreover, it follows that the Laplacian of the potential is equal to 0:

$$\nabla^{2}\Phi =0$$ 5

A Maxwell stress tensor can then be written as

$$\tau_{M}=\epsilon_{r}\epsilon_{0}\left(EE-\frac{1}{2}(E\cdot E)I\right)$$ 6

Due to the discontinuity in dielectric properties across the interface between the two fluids, a jump in the Maxwell stress **τ**_M_ arises. Following assumption (ii), the properties in the tangential direction are continuous so that there is only a jump across the interface (‘fluid 1 – fluid 2’) as indicated by [[ ]] and:

$$[[n\cdot \tau_{M}]]=\frac{1}{2}\epsilon_{r}\epsilon_{0}(E\cdot E)n$$ 7

A more complex scenario arises when the medium is a nonideal dielectric or poorly conducting: free charges generate tangential electrical stresses, producing fluid motion known as “leaky flows”.^63^ For our case, we can express the jump in scalar form since only a normal component exists, defining it as an electric pressure *P*_elec_:

$$P_{\mathrm{elec}}=\frac{1}{2}\epsilon_{r}\epsilon_{0}E^{2}$$ 8

Thus, for a film of permittivity ϵ_f_ = ϵ_1_ = ϵ_r,1_ ϵ_0_, the electric pressure can be estimated as

$$P_{\mathrm{elec}}\approx \frac{1}{2}\epsilon_{f}{\left(\frac{\Delta \Phi }{h}\right)}^{2}$$ 9

### Governing Equations—Hydrodynamics

For the hydrodynamic part, by using the following assumptions:

(i) bulk phases 1 and 2 are incompressible;
(ii) neglect inertial effects due to the small length scales;
(iii) neglect effects of gravitational forces,

it follows that the continuity and Stokes equation govern the problem:

∇·u=0 10

−∇p+∇·τ=∇·σ=0 11

where ***u*** is the velocity vector, *p* is the pressure and **τ** the bulk extra stresses. For Newtonian bulk phases with viscosities η_1_ and η_2_, the total mechanical stress (i.e., Cauchy stress tensor)**σ**_mech_ is

$$\sigma_{\mathrm{mech}}=-pI+2\eta D$$ 12

where ***D*** is the rate-of-deformation tensor and equal to (∇ ***u*** + (∇ ***u***)^*T*^)/2. Then, we can write the coupling between mechanical and electric stresses in a total stress tensor **σ**_T_ as

$$\sigma_{T}=\sigma_{\mathrm{mech}}+\tau_{M}=-pI+2\eta D+\tau_{M}$$ 13

where **τ**_M_ is given by eq 6. When the film becomes thin, there is additionally the disjoining pressure, which acts inside the thin film and accounts for the contributions due to intermolecular forces, which will be neglected here as films are relatively thick.

The conservation eqs 3, 5, 10, 11, and 12 must be solved for both the continuous oil film phase (fluid 1) and the dispersed aqueous phase (fluid 2). The interfacial properties then determine the boundary conditions and are needed to complete the problem.

### Boundary Conditions

The first boundary condition ensures continuity of the velocity across the interface: *u*_1_ = *u*_2_. Then, we can write the total stress (**σ**_T_) jump across the interface including the coupling between mechanical (**σ**_mech_) and electrical stresses (**τ**_M_), as

$$[[n\cdot \sigma_{T}]]=[[n\cdot \tau_{M}+n\cdot \sigma_{\mathrm{mech}}]]=\nabla_{s}\cdot \sigma_{s}$$ 14

where **σ**_s_ is the total interfacial stress, which can be written as **σ**_s_ = σ_αβ_ **I**_s_ + **τ**_s_, where σ_αβ_ is the interfacial tension, **I**_s_ = **I** – ***n*** ***n*** is the surface unit tensor, **τ**_s_ is the interfacial extra stress tensor and where ∇_s_ = **I**_s_ ·∇ is the surface gradient operator. Writing out the different bulk and interfacial stress contributions yields the following balance equation:

$$\tau_{M}\cdot n+(\sigma_{1}-\sigma_{2})\cdot n-(p_{1}-p_{2})n=\nabla_{s}\sigma_{\alpha \beta }-\sigma_{\alpha \beta }(\nabla_{s}\cdot n)n+\nabla_{s}\cdot \tau_{s}$$ 15

where the first term on the right-hand side represents the gradients in surface stress (Marangoni stresses), the second term accounts for the capillarity induced by curvature, and the third term represents the surface extra stresses related to interfacial rheological properties. This equation highlights the complex interplay that arises from the different contributions.^19^ The effects of interfacial rheology can be described by an appropriate constitutive equation, for instance the Boussinesq-Scriven constitutive equation^64,65^ for a purely viscous interface as

$$\tau_{s}=2\eta_{s}D_{s}^{d}+\kappa_{s}(\nabla_{s}\cdot u)I_{s}$$ 16

where η_s_ is the interfacial shear viscosity, κ_s_ the interfacial dilatational viscosity, and **D**_**s**_^**d**^ is the deviatoric part of the surface rate-of-deformation tensor given by **D**_**s**_ = (∇_s_ **u** ·**I**_**s**_ + **I**_**s**_ · (∇_s_ **u**)^*T*^)/2, so that **D**_**s**_^**d**^ = **D**_**s**_ – *tr*(**D**_**s**_)**I**_**s**_/2. Other constitutive equations are needed to describe more complex interfaces.

### Relevant Dimensionless Numbers

Different films are compared in terms of the relevant dimensionless numbers, namely the capillary number *Ca*:

$$Ca=\frac{\Delta P}{\sigma_{\alpha \beta }/R_{\mathrm{bw}}}$$ 17

where ΔP *R*_bw_ is the hydrodynamic driving force for drainage, with *R*_bw_ the radius of the bikewheel chosen as the characteristic length scale, and σ_αβ_ is the interfacial tension between the fluid phases resisting drainage. We also define an electrocapillary number *Ca*_e_ given by

$$Ca_{e}=\frac{\epsilon_{r}\epsilon_{0}E^{2}}{\sigma_{\alpha \beta }/R_{\mathrm{bw}}}$$ 18

where the numerator represents the electric driving force for drainage, i.e., the magnitude of the Maxwell stresses acting on the film. A Boussinesq number, which reflects the ratio of interfacial to bulk viscosities, is used to define interfaces having different resistance against drainage, as

$$Bq=\frac{\eta_{s}}{\eta R_{\mathrm{bw}}}$$ 19

## Materials and Methods

### Materials

N-hexadecane (99% Acros Organics) is used as the oil phase (fluid 1), which is filtered through a column of alumina oxide powder (Sigma-Aldrich) to remove polar components prior to use. As the aqueous phase (fluid 2), a solution of 0.6 M sodium chloride (NaCl purity of 99.99%, AlfaCesar) in Milli-Q water (resistivity 18.2 MΩ cm, Advantage A10, Merck Millipore) is used, both to mimic seawater (∼3.5 wt %) and have enough electrolyte concentration for the electric field measurements: at this concentration, the conductivity σ_2_ is O (1 S/m), and hence the charge relaxation time ϵ_2_/σ_2_ ∼ 10^–10^ s, which is much smaller than 1/ω in the experiments thus ensuring fast polarization. Also, $\sigma_{2}\gg \sigma_{1}∼O$ (10^–12^ S/m).

For the ‘simple interfaces’, the nonionic surfactant Span80 (Sigma-Aldrich, HLB = 4.3) is dissolved in the filtered hexadecane at a 1 wt % (≈0.8 %v/v) concentration ≈100 × CMC, forming sterically stable films.^66^ Despite the high concentration, no liquid crystals form, and the solution viscosity equals that of pure hexadecane i.e., η_1_ ≈ 3 mPa s, and hence only 3 × η_2_. Both solutions are saturated with the aqueous phase to prevent mass transfer during experiments.

For the ‘complex’ interfaces, asphaltenes were provided by the Ugelstad Laboratory in NTNU (Trondheim, Norway) and were obtained by precipitation from North Sea crude oil using *n*-hexane.^15,67^ Asphaltenes are dispersed at a concentration of 1 mg/mL in toluene (VWR Chemicals, AnalaR Normapur >99.5%) in order to specifically explore the colloidal nature of asphaltenes: according to the Yen-Mullins model,^68^ at this concentration asphaltenes are present as nanoaggregates. Interfacial rheology and thin film balance experiments used asphaltene layers created by spreading asphaltenes from a good solvent onto the liquid–liquid interface, where the oil phase is asphaltene-insoluble.^15^ The volume of solution spread varies across different experiments, as the interfacial area sizes are different, but are always spread to achieve the same surface coverage to ensure a meaningful comparison. This approach avoids adsorption effects, ensures better surface coverage control, yields mechanically strong interfaces immediately (without hours of aging), and reduces spontaneous emulsification. Figure S1 illustrates that while asphaltene-in-toluene interfaces develop μm-sized droplets over 30 min, hexadecane-water interfaces remain largely droplet-free. This distinction is critical, as droplet presence significantly impacts interfacial rheology and film drainage, especially under electric fields. It has been shown that some solvent (toluene) can still be entrapped within the nanoaggregates even after long waiting times,^69,70^ yet this most likely comes closer to reflecting real asphaltene interfaces as recently argued by.^71^ While more realistic conditions involving partial solubility merit study, this work focuses on densely packed layers to isolate the mechanical properties affecting drainage and electrocoalescence.

A commercial demulsifier (fatty acid alkoxylate ester, RSN 6.1) used in^38,39^ is dissolved in xylene (VWR Chemicals >96%) at 0.1–10 mg/mL for rheology experiments. It is added dropwise (50 μL) to the top oil phase, with concentrations reported in ppm based on total oil volume (0.5–50 ppm). For thin film drainage experiments, the demulsifier is added from the aqueous phase at similar concentrations.

The Wilhelmy plate method was used to measure the static interfacial tension of all the materials used here, namely (i) 1 wt % Span80 solution in hexadecane, (ii) asphaltenes (densely packed interface), (iii) demulsifier (5 ppm solution), as reported in Table 1. Measurements were carried out at 23 ± 0.5 °C with a KSV Nima balance.

**Table 1:** Equilibrium Interfacial Tension Values for the Different Surface-Active Materials Used in This Study Measured at *T* = 23 ± 0.5°C

	Span80	asphaltenes	demulsifier
σ_αβ_ [mN/m]	3.1 ± 0.2	35.5 ± 0.5	2.4 ± 0.3

### Interfacial Rheology

Interfacial shear rheology measurements are performed using a double wall-ring (DWR) geometry^72^ on a DHR-3 rheometer (TA Instruments). The setup includes a Teflon cup placed on the Peltier plate and a Pt–Ir ring attached to the rheometer’s upper part. Following,^15^ the aqueous subphase is added to the cup, and the ring is positioned at the surface. The oil phase is carefully layered on top, and the system is thermally equilibrated for 5 min. The asphaltene solution is then spread dropwise onto the interface with a Hamilton μL-syringe to achieve the desired surface concentration.

Oscillatory time sweeps are carried out to monitor the temporal evolution of the shear viscoelastic moduli *G*’ and *G*” and are therefore initiated immediately at a fixed strain amplitude of 0.02% (within the linear viscoelastic response regime) and a frequency of 2 rad/s. Demulsifiers are introduced at *t* = 1000 s via dropwise addition to the oil phase at concentrations of 0.5–50 ppm. All experiments are conducted at 23 ± 0.1 °C and at least three replicates are obtained to verify the general trends.

### Electric Thin Film Balance

To study electro-coalescence at a thin film scale, we combine the dynamic thin film balance technique (DTFB) with precise pressure control^73,74^ and a patch clamp technique previously used to study lipid bilayers^75^ as a way to impose electric fields across thin films. Figure 2 depicts a schematics of the main components as described below.

**Figure 2:**
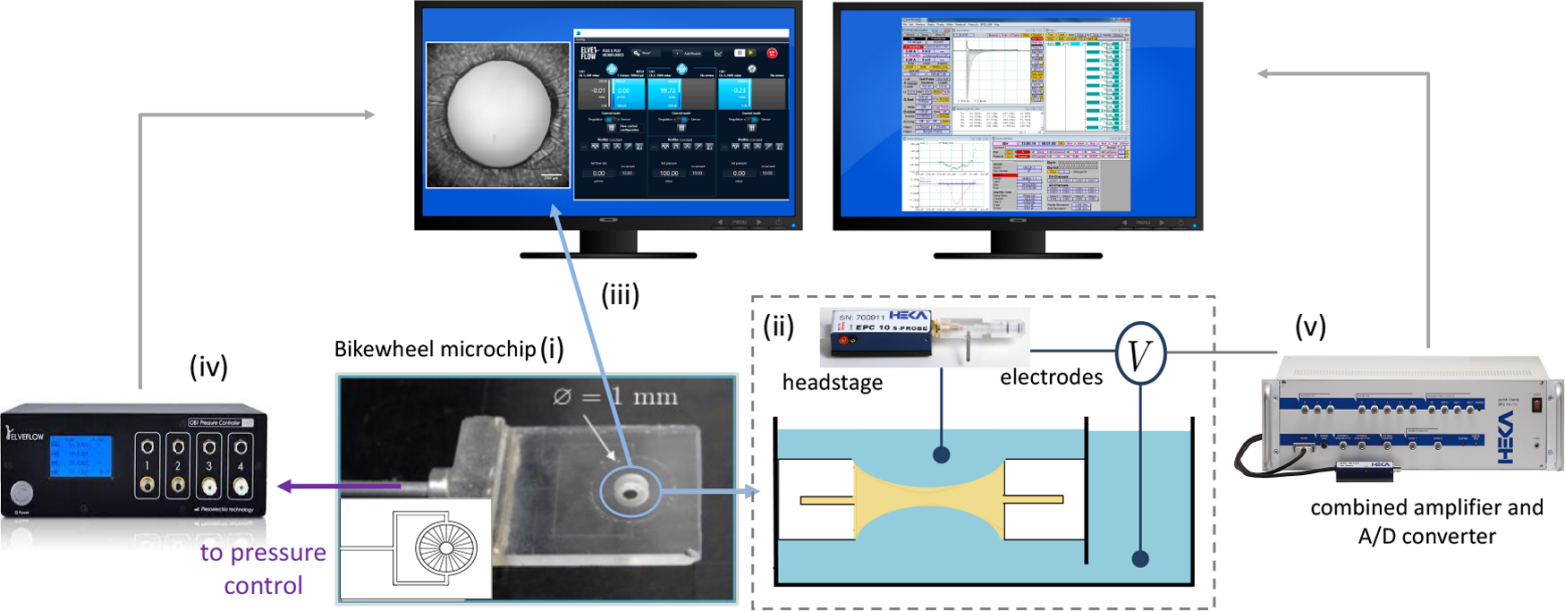
Schematics of the electric thin film balance setup and its main components, including (i) bikewheel microfluidic chip, (ii) Faraday cage, (iii) upright microscope, (iv) Elveflow pressure control, (v) Heka Patchclamp amplifier. The cartoon illustrates the side view of an oil-in-water film formed inside the chip.

An emulsion film (oil film surrounded by an aqueous phase) is created in a bikewheel microfluidic chip. The design of the chip is based on the work by Cascão-Pereira et al.:^76^ it features a central hole with a diameter of 1 mm and thickness 400 μm, and 25 radially distributed spokes (width of 45 μm and depth of 20 μm) around its perimeter to allow for homogeneous drainage of the film. The chip is custom-fabricated from glass using photolithography (Micronit Microfluidics), and undergoes surface treatments to ensure oil film pinning at the sidewall. First, the outer surfaces are hydrophilized by sonication in an NaOH solution. Then, the internal channels and the surface around the 1 mm hole (contacting the oil film) are hydrophobized via silanization with OTS. Detailed procedures are provided in ref 73.

For the experiments, first this chip is filled with the oil phase and placed inside a POM (polyoxymethylene) chamber. For surfactant-films, the bulk oil phase is the surfactant solution, whereas for asphaltene experiments, pure hexadecane is used, with asphaltene solution spread directly onto the film interfaces. Subsequently, the 0.6 M NaCl aqueous phase is carefully added to both the bottom and top chambers to prevent air entrapment beneath the chip. The DTFB setup is placed under an upright microscope (Nikon Eclipse FN1) and a 10× long-working distance objective is used to image the film in reflection mode under a specific wavelength (λ = 508 nm) for interferometry. The film is monitored with a 16-bit grayscale Hamamatsu ORCA-Flash4.0 CMOS camera. Images during drainage are recorded at 1 fps and for film rupture experiments at 10 fps. The chip is connected with rigid Teflon tubing to an Elveflow MK3+ pressure control system, which ensures the pressure inside the film can be precisely controlled (±1 Pa) with temporal evolutions to mimic coalescence.^74^

To study the effects of electric fields, the setup developed by Beltramo et al.^75^ to study ion-transport across lipid bilayers is used. Silver electrodes are placed on each side of the POM chamber to apply a potential difference ΔΦ across the film. Electrical isolation between the top and bottom sides of the film is achieved using high-viscosity vacuum grease around the edges of the glass chip, and is verified by the minimal current ∼O ­(10^–12^ A) measured with an oil film in place. The electrodes are connected to a S-probe headstage and enclosed in a Faraday cage to minimize external noise. The headstage interfaces with a patch clamp amplifier (EPC10, Heka), which is connected to a computer for input control (e.g., applied voltage) and output recording (e.g., current) using Patchmaster software (Heka). The maximum voltage that can be applied here is 2000 mV and maximum current measured is 100 nA.

In drainage experiments, film thickness is typically calculated from interferometry using the Scheludko equation.^53^ While effective for foam films due to the significant refractive index difference between liquid and gas, this method is less precise for oil–water systems owing to their low refractive index contrast, i.e., *n*_1_ = 1.434 and *n*_2_ = 1.337. To address this, here we use capacitance measurements to calculate the thickness of the films.^75^ Briefly, the system comprises a hydrophobic core with a low dielectric constant (ϵ_r,1_ = 2.08 for hexadecane) surrounded by a medium of high dielectric constant (ϵ_r,2_ = 80 for the NaCl solution) as depicted in Figure 2, forming a capacitor. The capacitance of the film *C*_f_ is related to its area *A*, thickness *h* and dielectric constant ϵ_f_ as

$$C_{f}=\epsilon_{f}\left(\frac{A}{h}\right)$$ 20

By measuring the capacitance and the film area from the images, the film thickness can be calculated. This method has been applied to study emulsion film thicknesses, such as polymer-stabilized,^77^ and to calculate the dielectric constant for crude oil films with thicknesses assessed by interferometry.^51^ Here, the dielectric constant used in the thickness calculation assumes a pure hexadecane film, and we will discuss the validity of this assumption later on. The capacitance technique involves applying a small-amplitude AC field (frequency 1 kHz, an amplitude of 10 mV and an offset of 10 mV). We verified that these parameters do not significantly alter the drainage behavior compared to measurements without capacitance monitoring.

#### Experimental Procedure

Due to the complex interplay of hydrodynamics, capillarity, intermolecular forces, and interfacial stresses, deriving a complete pressure balance for this system is challenging. However, we present a simplified model to highlight the relevant contributions during the experiments (see also Supp. Info S2). These pressure balances build on an earlier framework^78^ for polymer foam films, extended here to account for complex interfaces and the additional contribution of the electric field.

Three types of experiments were carried out, namely:

(I) drainage under constant pressure (Δ*P*) while monitoring the capacitance;
(II) drainage under constant pressure (Δ*P*) and an applied DC voltage (ΔΦ);
(III) increasing the DC voltage in steps (ΔΦ) for stable films.

In type (I) and (II) experiments, the first step involves determining the film’s equilibrium pressure *P*_eq_, the pressure at which the film is in mechanical equilibrium and neither drains nor thickens. This corresponds to the point where the first interference fringes appear (at thicknesses of about 10^3^ nm) and at this point the pressure balance is

$$P_{2}+P_{L}=P_{1}=P_{\mathrm{eq}}$$ 21

where *P*_1_ is the pressure inside the meniscus of the liquid film, *P*_2_ is the pressure of the surrounding aqueous phase, and *P*_L_ is the Laplace pressure of magnitude ≈2σ_αβ_/*R*_bw_ due to the curvature in the “Plateau border” of the film. Then, in experiments type (I) a pressure *P*_0_ < *P*_eq_ is imposed inside the film, which sets the drainage pressure step Δ*P* = *P*_eq_ – *P*_0_. Then, the pressure balance becomes

$$P_{2}+P_{L}=P_{1}-\Delta P+P_{\sigma_{s}}$$ 22

Once the film starts draining, we include an additional pressure term, *P*_σ_s__, to account for the effects of interfacial stresses that resist drainage. These stresses arise from effects such as Marangoni stresses or interfacial elasticity.

No other pressure contributions arise at this stage, since the films are too thick for intermolecular forces to play a role and hydrodynamic pressure has not build up yet. In type (II), there is an additional contribution due to the electric field:

$$P_{E}+P_{2}+P_{L}=P_{1}-\Delta P+P_{\sigma_{s}}$$ 23

where *P*_E_ is the electric pressure given by eq 8, which will exert a compressive force on the film.

In both cases (I) and (II), the experiment starts when *P*_0_ is applied to the ‘thick’ film: this sets *t*_0_. It is worth noting that this is different than the drainage time *t*_d_, which starts when the thin film starts to expand.

Then, when a thin film forms, the pressure balance becomes much more complex and depends on the interplay between hydrodynamics, capillarity, interfacial stresses and electric fields^19^ as

$$P_{E}+P_{2}+P_{L}=P_{1}-\Delta P+P_{\sigma_{s}}+P_{H}(h,r)+\Pi_{\mathrm{disj}}(h,r)+\frac{\sigma }{2r}\frac{\partial }{\partial r}\left(r\frac{\partial h}{\partial r}\right)$$ 24

where *P*_H_(*h*, *r*) is the hydrodynamic contribution; Π_disj_(*h*, *r*) is the disjoining pressure; and the last term is a contribution due to local changes in film curvature. The disjoining pressure is defined as the sum of the equilibrium intermolecular forces that act between the two surfaces of the film and entail both attractive (due to van der Waals forces) and repulsive contributions (electrostatic and steric).^53,79^ Here, we can neglect the electrostatic repulsion, so that Π_disj_ = Π_vdW_ + Π_steric_. The draining film will then either rupture or remain stable depending on the complex interplay between all these contributions.

Type (III) experiments are carried out for stable thin films only, i.e., for those that did not break during drainage in (I) and (II) and remain stable for at least 15 min after that. For these, the pressure inside the film is kept at *P*_0_ and the experiments begin at *t*_0_ with ΔΦ = 0 mV; then ΔΦ is increased stepwise every 10 s. The current *I* is recorded throughout the experiment and serves as a second indicator for film rupture.

### FEM Simulations

Numerical simulations are carried out to demonstrate the effect of electric fields in thin film drainage at constant pressure drop ΔP and under constant electric potential ΔΦ, akin to experiment type (II) in hydrodynamics-dominated regime. The momentum and mass balance (eqs 10 and 11), and the Laplace equation for the electric potential (eq 5) form a set of coupled equations which are solved using the finite element method. Axisymmetric simulations are performed, whereby only a quarter of the full domain as depicted in Figure S3 is discretized using appropriate symmetry and boundary conditions. We use iso-parametric, triangular *P*_2_/*P*_1_ (Taylor-Hood) elements for the velocity/pressure fields, as well as *P*_2_ elements for the potential field. Adaptive meshing is used when the elements become too distorted and the mesh is refined near the thinnest parts of the film. More details about the numerical simulations, such as parameters used and mesh- and time-convergence can be found in the Supporting Info.

We chose the parameters in the simulations in such a way that a qualitative comparison to the experiments could be made, and all the results are presented in dimensionless form. Specifically, the *initial* capillary number and electric capillary number: are *Ca* = 3.27, *Ca*_e_ = 0.8, which are in the range of the experimental results. The minimal initial thickness is assumed to be *h*(*t*_0_)/*R*_bw_ = 0.02, which corresponds to a dimensionfull initial thickness of 10 μm. As discussed in section Boundary Conditions, the interplay between all the different contributions in the interfacial stress balance is too complex and not fully elucidated yet, thus only the effects of surface tension and surface viscosity will be included as a stress boundary condition using eq 16. Simulations are performed for two cases: an interface that cannot support shear stresses (η_s_ = 0) and an interface that has strong resistance against shear stresses (η_s_ = 1000), where the latter case effectively tangentially immobilizes the interface.^19^

## Results and Discussion

First, we examine nonionic surfactant films and explore the effects of the electric fields on a ‘surface active’ interface. We start by analyzing the drainage behavior (type I experiments), then proceed to interrogate the effect of electric fields during drainage (type II) and finally their impact on stable thin films (type III). We then discuss the mechanisms leading to film rupture in each case also in light of FEM simulations. Finally, we proceed with the investigation of asphaltene-laden films, emphasizing the differences between simple and rheologically complex interfaces. At the end, we also interrogate the effect of chemical demulsifiers and their synergistic effect with electric fields.

### Surfactant Films

#### Drainage

We first discuss the typical behavior observed for a surfactant film draining under a constant pressure (type I). Figure 3 shows interferometric images of a film draining at Δ*P* = 5 Pa alongside capacitance monitoring. Initially, a small dimple forms, grows, and becomes asymmetric. Black spots then nucleate and expand until a uniform black film forms. This behavior aligns with that expected for nonionic surfactant films at high concentrations, where drainage begins with hydrodynamic dominance and transitions to stability governed by intermolecular forces.^19,80^ In the sequence shown in the figure, a stable thin black film was formed, but in about 30% of the experiments with this Δ*P* the film ruptures when black spots start to nucleate. Qualitatively similar drainage behavior was observed for the entire pressure range investigated (5–50 Pa), corresponding to *Ca* = 1∼10. Dimples formed at *Ca* > 1 and indicate that hydrodynamic forces dominate the flow. Drainage times *t*_d_ are shown in Figure 5 (black circles) and decrease with applied Δ*P*, as expected. The relatively slow drainage (on the order of minutes) is attributed to the high surfactant concentration, which enhances Marangoni stresses, as evidenced by the asymmetric films, resisting drainage.

**Figure 3:**
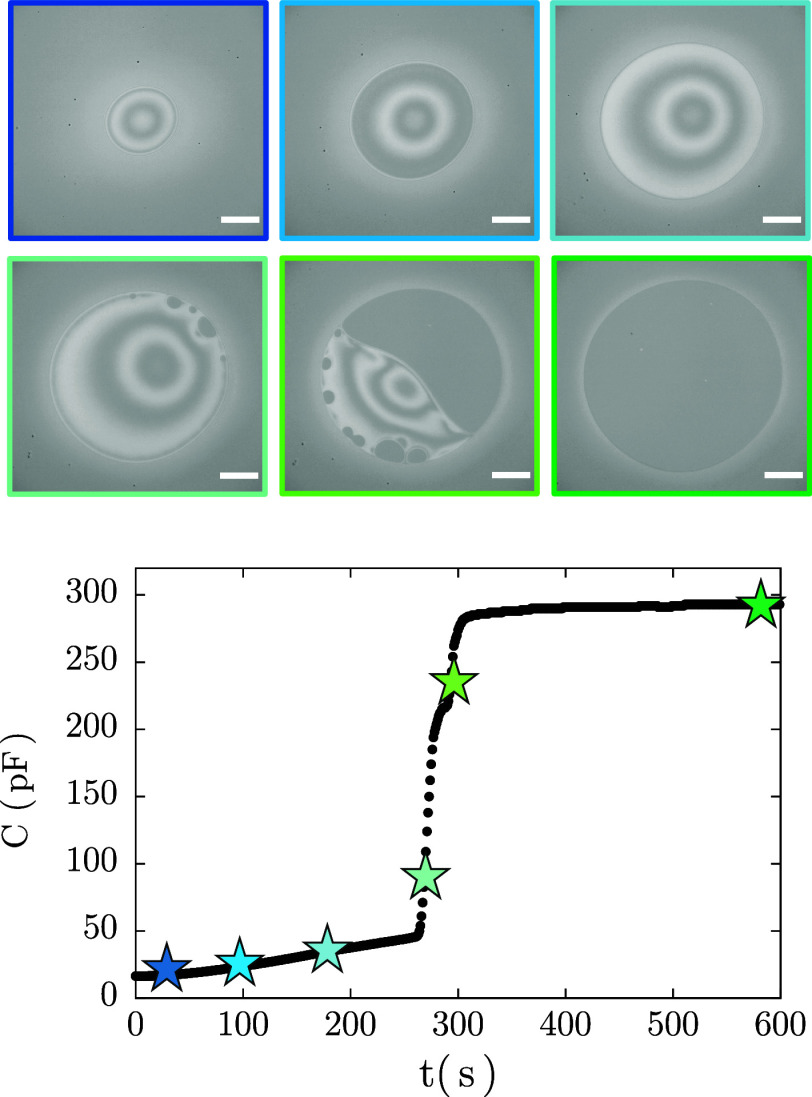
Drainage of a Span80 surfactant film at Δ*P* = 5 Pa (*Ca* = 0.8): capacitance measured over time with representative images of the drainage process. The color of the image outlines corresponds to the stars in the plot. Scale bar: 100 μm.

The stages of drainage are evident in the capacitance curve. Initially, for a thick film (∼10^3^ nm) the capacitance is *C*_i_ = 16.0 ± 0.5 pF. During early drainage, as a dimple forms and the film diameter ranges from 100–200 μm, the capacitance increases only mildly. However, once black spots nucleate, the capacitance increases significantly reflecting the dramatic thickness reduction. Once a stable thin film forms, the capacitance stabilizes at *C*_eq_, which has a value about 20× larger than the initial one. This equilibrium capacitance (stemming from data as in Figure S7) is then taken to calculate the equilibrium film thickness *h*_eq_ via eq 20. Figure 4 shows *h*_eq_ as a function of *Ca*, with *h*_eq_ = 9.4 ± 2.6 nm, consistent with typical reported film thickness for Newton black films (NBF).^81^

**Figure 4:**
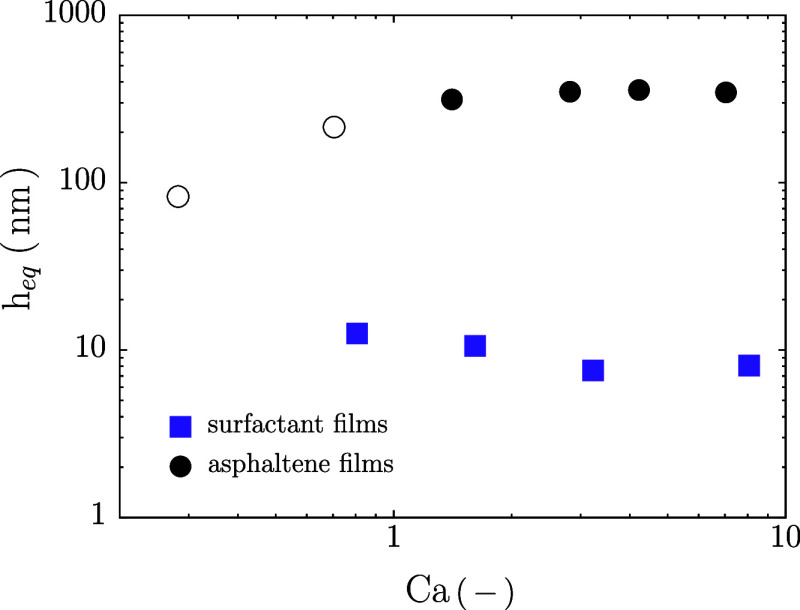
Equilibrium film thickness (*h*_eq_) of surfactant (black circles) and asphaltene films (purple squares) as a function of capillary number *Ca*, calculated from the film equilibrium capacitance *C*_eq_ with eq 20. Empty symbols correspond to experiments in which the thin film area *A*_f_ is small compared to the total film area *A*_bikewheel_, so that the film capacitance is small compared to the total measured capacitance (and thus rendering thickness calculation less precise).

Politova et al.^66^ reported black film thicknesses of *h*_eq_ ≲ 10 nm for Span80 in hexadecane emulsion films, consistent with steric repulsion between surfactant tails. While they observed stable thick films (>200 nm) at very high Span80 concentrations (≫ CMC) with NaCl, we did not, likely due to our use of saturated phases to prevent mass transfer. Similarly, Narayan et al.^82^ inferred stable films in Span80-stabilized droplet collisions, attributing stability to steric and structural forces from inverse micelles, but film thickness was not accessible in these experiments.

We examine next the effect of electric fields on the dynamic drainage behavior (type II) by measuring drainage times. In these experiments, the *initial Ca*_e_ varied from 0.003–1; as it indirectly depends on film thickness, throughout the drainage it increased up to ≈5. Figure 5a compares results at different ΔP (black data points) with films drained under an additional applied potential ΔΦ = 100 mV. Drainage is faster with ΔΦ, and the nearly Δ*P* - independent drainage times suggests that the electric field is dominating the drainage behavior. This is also evidenced in Figure 5b, which shows the drainage time decreasing linearly with increase in applied voltage ΔΦ for a given Δ*P*.

**Figure 5:**
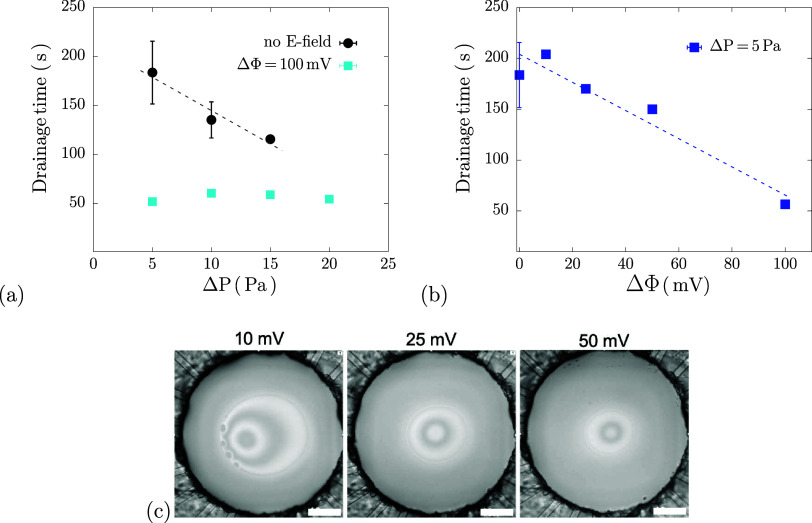
Effect of electric fields on surfactant film drainage times: (a) Comparison of drainage with and without electric fields at different Δ*P*; (b) Drainage times for a Δ*P* = 5 Pa, different applied ΔΦ. Dashed lines are linear fits to the data. (c) Final frames before rupture of Span 80 films for different applied voltages corresponding to data in 5b. Scale bars = 200 μm.

The final frames before rupture (Figure 5c) for different applied ΔΦ reveal no stable NBF films formed in type II experiments. Faster drainage with increasing ΔΦ is due to rupture occurring earlier, still in the hydrodynamic regime. Specifically, film rupture occurs at the point of black spot formation for ΔΦ = 10 mV, while for 25 and 50 mV the films rupture still as a dimple.

This effect can be explained by considering the electric pressure *P*_elec_ acting on the film using eq 9 as shown in Figure 6. Assuming rupture occurs just before or after black spot formation, at *h* = 45 ± 5 nm,^66^ we can then estimate *P*_elec_ ≈ 10–20 Pa for a film under 50 mV and *h* ∼ 50 nm. This ‘critical electric pressure’ is in the same order of magnitude of the other components in the pressure balance for this system in eq 23, namely the drainage pressure Δ*P* = 5 Pa and the Laplace pressure *P*_L_ = 12.4 Pa, in this regime dominated by hydrodynamics. While these *P*_elec_ values rely on several strong assumptions (see section Theoretical Background), they provide useful order-of-magnitude comparisons between pressure contributions.

**Figure 6:**
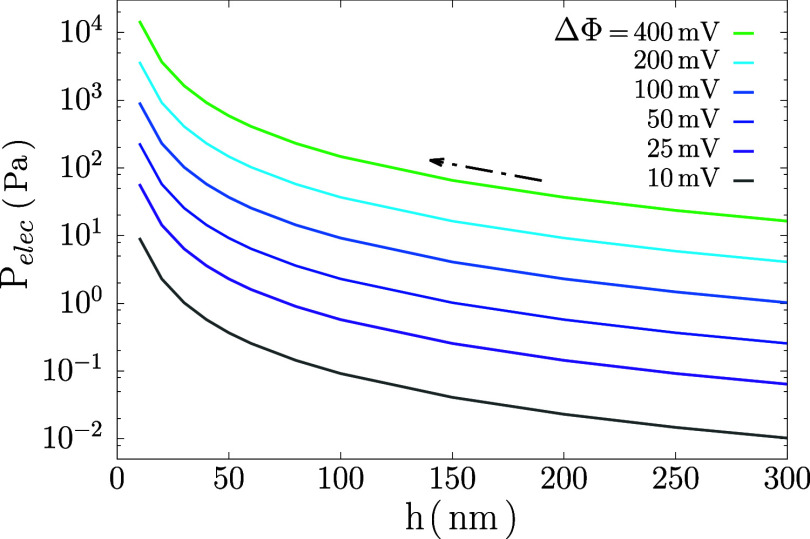
Electric pressure as a function of film thickness for different applied ΔΦ; arrow indicates the drainage direction of decreasing thickness.

#### Rupture

Our previous results demonstrate that electric fields destabilize surfactant-stabilized films. To explore the underlying mechanisms, we investigated stable NBF films (from Figure 3) using type III experiments, where the voltage was incrementally increased until rupture (Figure 7 and Movie S1). In Figure 7a, the critical voltage for rupture is evident from the sharp current spike at *t* ≈ 93 s, corresponding to 175 mV. Rupture was also visualized, as can be seen in Figure 7c. Interestingly, film rupture occurs 2–3 s after the voltage increase, a delay unrelated to charge motion, as charge relaxation time scales are ∼10^–10^ s. A gradual current increase preceding rupture, highlighted in the inset of Figure 7a can be observed and this suggests rupture may involve electroporation, similar to phospholipid membranes.^56,83^ In this mechanism, electric fields create pores in the bilayer. If line tension is insufficient to close the pore, it expands, leading to rupture when its radius approaches the value of the film thickness. The electric field strengths in our experiments (10–100 kV/cm or 10^6^–10^7^ V/m; see Figure S8b) align with values observed in similar studies. Anklam et al.^49^ reported a comparable current increase during voltage ramp experiments in polymer-stabilized films, attributed to electroporation, despite their films being even ∼10× thicker.

**Figure 7:**
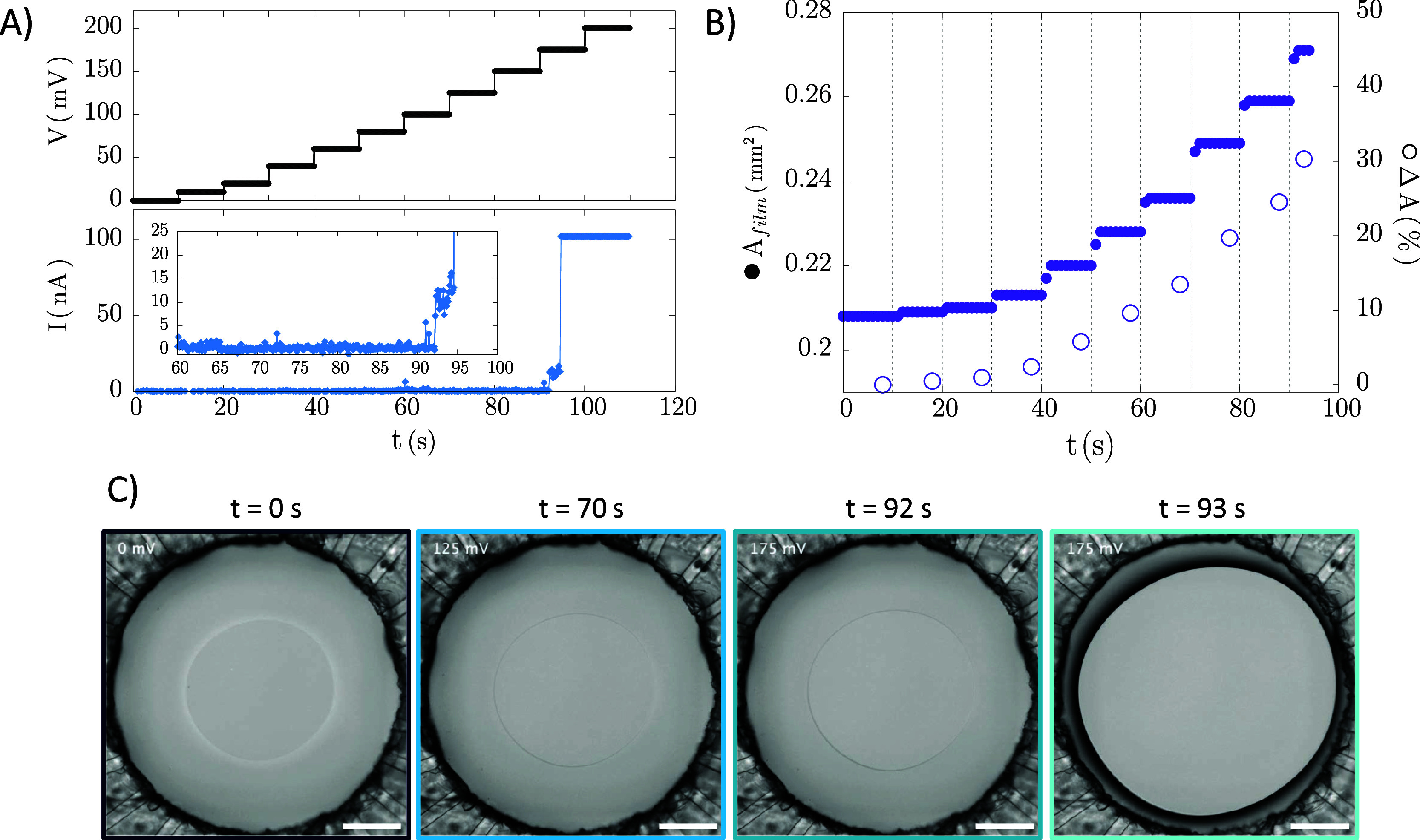
Voltage steps experiment (type III) on a stable surfactant thin film that was drained at a constant Δ*P*: (a) applied voltage *V* and measured current *I* as a function of experiment time; (b) Film area (filled symbols) and area change (empty symbols) as a function of experiment time; (c) Representative images of the experiment - the last two images correspond to the last frame before rupture and first frame after rupture, respectively. The scale bar in the images corresponds to 200 μm.

Stable thin surfactant films typically ruptured at applied voltages between 175 to 250 mV. No clear correlation between film thickness and critical rupture voltage has been established, warranting further investigation. Importantly, films remained intact if the critical voltage was not reached (see Figure S9): in the first experiment (black squares in (a), top row in (b)) the voltage was increased to 200 mV and the film remained stable. In fact, upon setting the voltage back to 0 mV, the film shrank and fully recovered, showing that the effect of the electric pressure is completely reversible. In a subsequent experiment (cyan triangles in (a), bottom row in (b)), the same film was subjected to higher ΔΦ and broke at 250 mV.

Notably, the critical voltages observed here are much higher than the ones needed to rupture draining films. Referring to Figure 6, for NBF with *h* ≈ 10 nm, the electric pressure at these critical voltages ranges from 2800 to 5800 Pa, over 2 orders of magnitude higher than for draining films. It is worth mentioning that we use a dielectric constant of the film equal to that of hexadecane since the specific value for Span80 is not known, but despite the fact that these thin films are dominated by the surfactant layers, such differences in ϵ_f_ cannot account for this disparity. The stability of these films under compressive electric pressures up to at least 2 kPa is attributed to their disjoining pressure, most likely due to steric repulsion between surfactant tails. None of the other components in the pressure balance given in eq 24 will have such a large magnitude. Disjoining pressures in surfactant-stabilized foam films beyond CMC are typically a few kPa,^84^ consistent with oil-soluble surfactants like Span80, as reported by Liu et al.^85^ Their droplet–droplet experiments under electric fields, inspired by Anklam et al.,^77^ found Π_disj_ ∼ kPa. Hence, at these small thicknesses, the disjoining pressure determines the critical electric pressure and, consequently, the critical voltage for rupture.

In all experiments, the films exhibit two distinctive features during stepwise increases in ΔΦ: (i) the film expands in steps, and (ii) the rim progressively darkens. Figure 7b shows the evolution of film area, both in absolute terms (*A*_film_, filled symbols) and as a percentage change (Δ*A*, empty symbols). Vertical dashed lines mark voltage increments, revealing stepwise area increases, with the film adjusting to each new ΔΦ within ∼1 s. Remarkably, the film area increases by up to 30% until rupture. Next, we interrogate whether the film thickness is also changing alongside the area changes.

As previously discussed, accurately determining the film thickness for emulsion films via interferometry is challenging. Still, intensity changes provide valuable insights. Figure 8a shows that the intensity at the film center remains constant until rupture suggesting that the thickness does not change. While the profiles shown in Figure 8b are largely uniform across the diameter of the film, two local minima appear at ∼30 and ∼570 μm from the center, corresponding to the thin film’s edges. In Figure 8c, which focuses on the shaded area in Figure 8b and curves are shifted vertically by arbitrary units to enable better visualization of the data, it is seen that the local minima intensify with increasing voltage indicating a local decrease in thickness, and their lateral shift reflects film area expansion.

**Figure 8:**
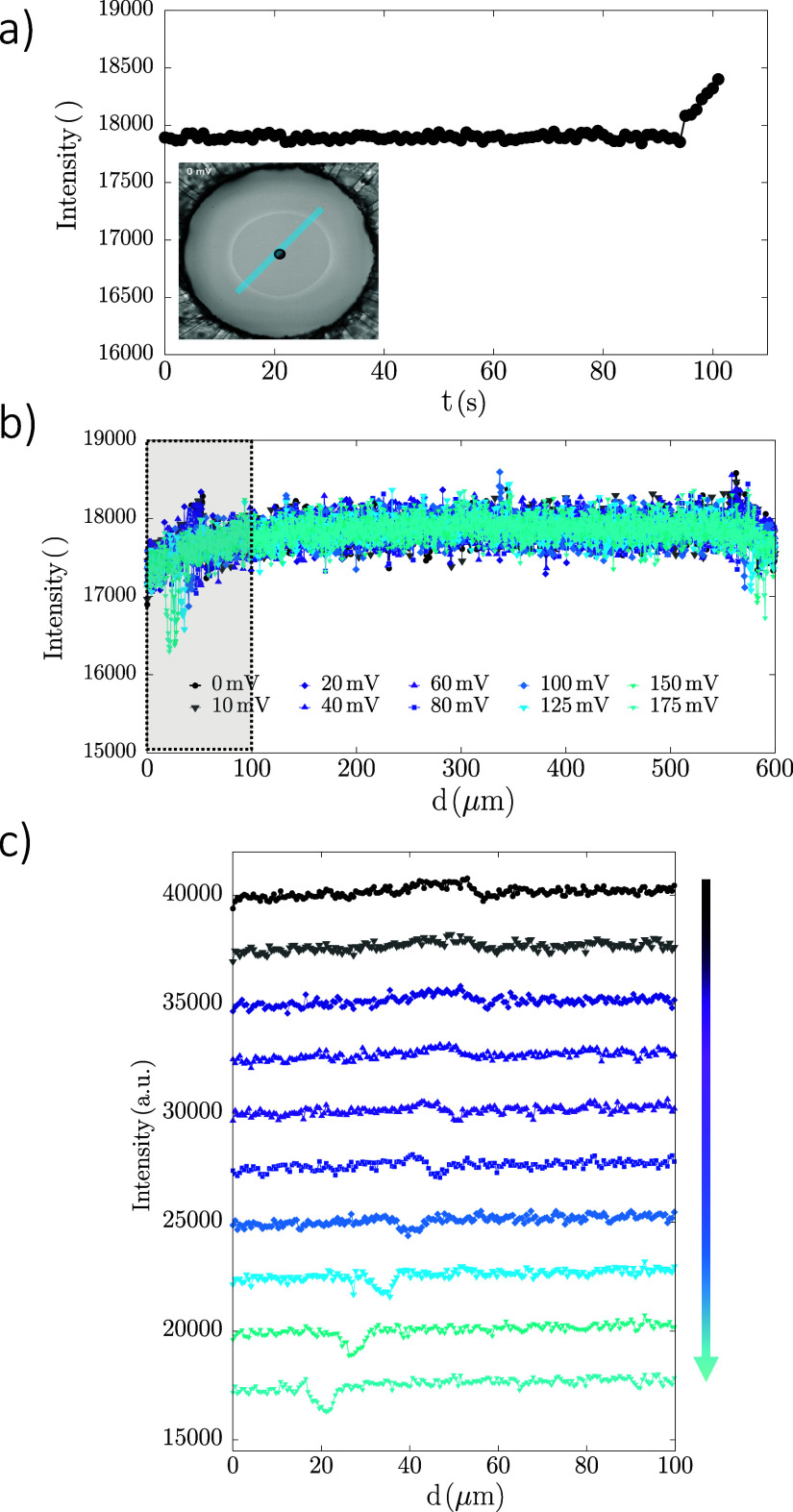
Rupture of surfactant films (a) Intensity measured at the center of the film over experimental time; (b) Intensity profile across the film diameter (as indicated by the blue line in (a)) measured for different voltage steps; (c) zoomed in view of the same data on the gray-shaded area of (b) for better information on what happens at the rim - curves are vertically shifted by arbitrary units only to avoid overlap of many curves. The arrow indicates the direction of increasing voltage.

These observations suggest that even small applied ΔΦ potentials can generate significant local electric field strengths, which get amplified in thinner regions. As *P*_elect_ ∝ *h* ^–2^ (see Figure S8a), areas of smaller thickness experience greater compressive forces, ultimately leading to rupture. In fact, from the images immediately after breakup in Figure 7c, we can note that the film indeed breaks along its circumference.

### FEM Simulations

FEM simulations were carried out to better illustrate how the electric field gets locally amplified in different draining films. These simulations correspond to experiments of drainage under constant pressure and constant applied potential DC (type II), here with *Ca* ≈ 3 and initial *Ca*_e_ = 0.8. Results are shown in Figure 9 for (a) interface that has no shear resistance and (b) a film with higher shear resistance, where the color gradient indicates the electric field strength *E* inside the film. Note that the values shown in this figure are nondimensionalized according to the parameters given in the Supporting Info. Figure 9c shows a slice through the same films to highlight thickness changes along its diameter, respectively at the initial condition and just before rupture for (a) and (b), as indicated by the colored outlines.

**Figure 9:**
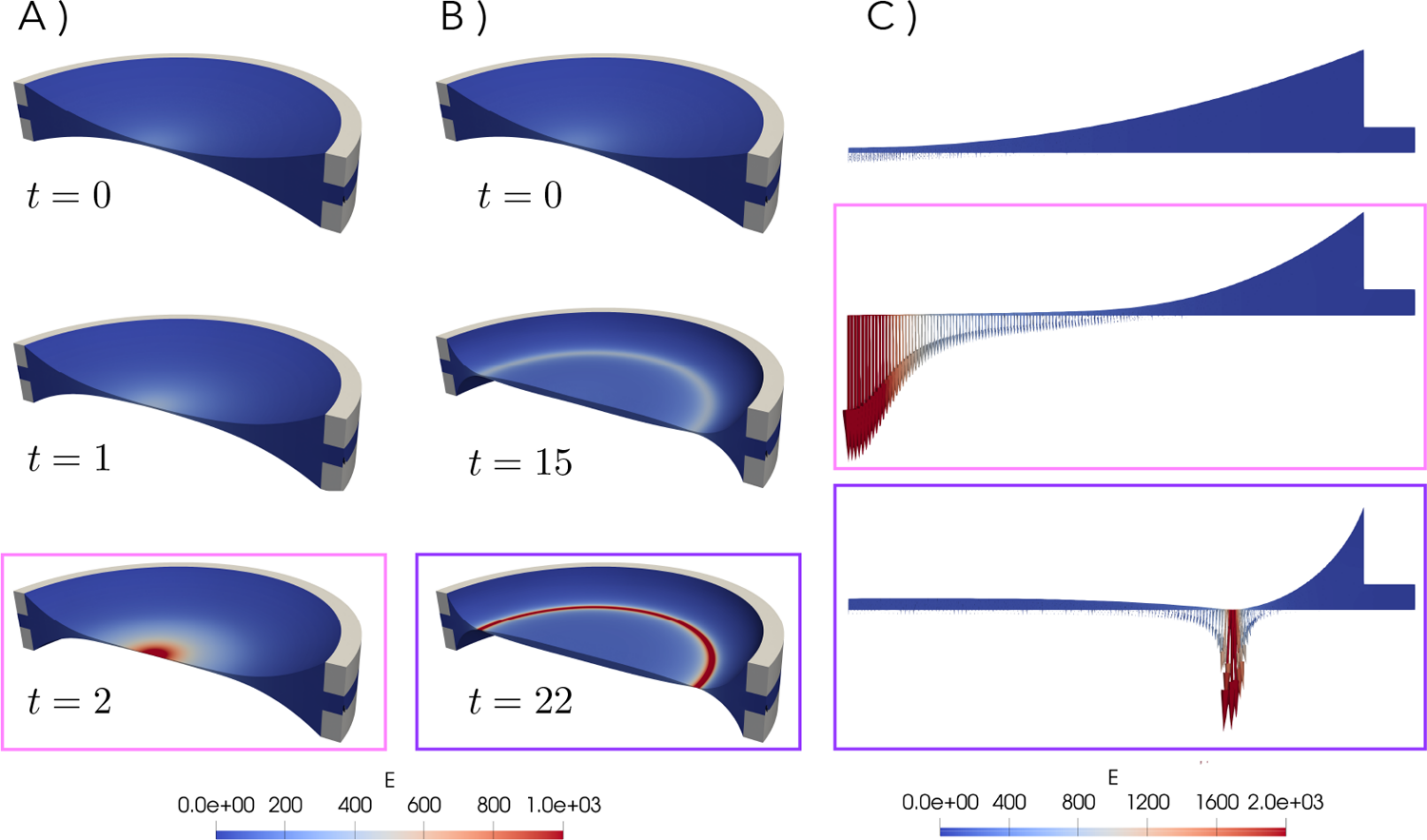
Representative FEM drainage results: (a) planar film with snapshots taken at *t* = 0, 1 and 2; (b) slightly dimpled film, snapshots taken at *t* = 0, 15 and 22; (c) profile of the same films at *t* = 0, and last moment before rupture for planar and dimpled film, respectively. The color scale reflects the magnitude of electric field strength. Initial conditions of the simulations correspond to *Ca* ≈ 3, *Ca*_e_ = 0.8, and (a) *Bq* = 0 and (b) *Bq* = 1000.

It can be seen that film (a) drains as a planar film, while (b) forms a slight dimple when draining. It is striking that the electric field gets localized at different locations for either films, namely at the center for the planar film and at the rim for the dimpled films. In (b), a slightly dimpled film arises during drainage, which is enough to lead to amplification of the electric field along the rim of the film. This is in agreement with what we observe from the experiments: a film that is slightly thinner along its rim and how the electric field gets locally amplified.

These results show very clearly that the electric field strength is dramatically amplified in regions of smaller thickness, corroborating our previous experimental observations. Given the complexity of the interplay between different surface stress contributions, a direct comparison between experimental and numerical results is beyond the scope of this work. As it is challenging to model and describe such complex systems, we will investigate experimentally the case of “rheologically active” interfaces in the next section.

### Asphaltene Films

Following the structure of section Surfactant Films, we first present dynamic drainage experiments and then discuss the rupture of stable films, including effects of demulsifier addition. As explained earlier, we use insoluble asphaltene films so that we retain the same bulk phases as before, namely hexadecane as the film phase and 0.6 M NaCl aqueous solution as the external phase, thereby altering only the interfacial properties.

#### Drainage

Asphaltene films exhibit markedly different drainage behavior compared to surfactant films. Figure 10 shows capacitance data and representative images for a draining film at Δ*P* = 50 Pa (*Ca* = 0.7). Initially the thick film value, *C*_i_ = 16.5 ± 0.6 pF, matches that of the surfactant films (Figure 3), indicating that bulk properties dominate, as expected. However, from the onset of drainage, the film appears highly heterogeneous due to asphaltene nanoaggregates at the interface. Unlike surfactant films, no dimples form, as evidenced by the absence of concentric patterns. Instead, a planar film develops, expands slowly, and then stabilizes for extended periods. In most experiments 15 min of waiting time were allowed until the next step, but we have observed the films to be stable over an hour (maximum observed duration). Figure S10a shows flat capacitance curves during the final minute for various drainage pressures, confirming no thickness or area changes. Films expanded to the full diameter of the bikewheel hole under maximum Δ*P* = 500 Pa. The investigated Δ*P* range corresponds to *Ca* ≈ 0.3–7, which is in the same range as for the surfactant films.

**Figure 10:**
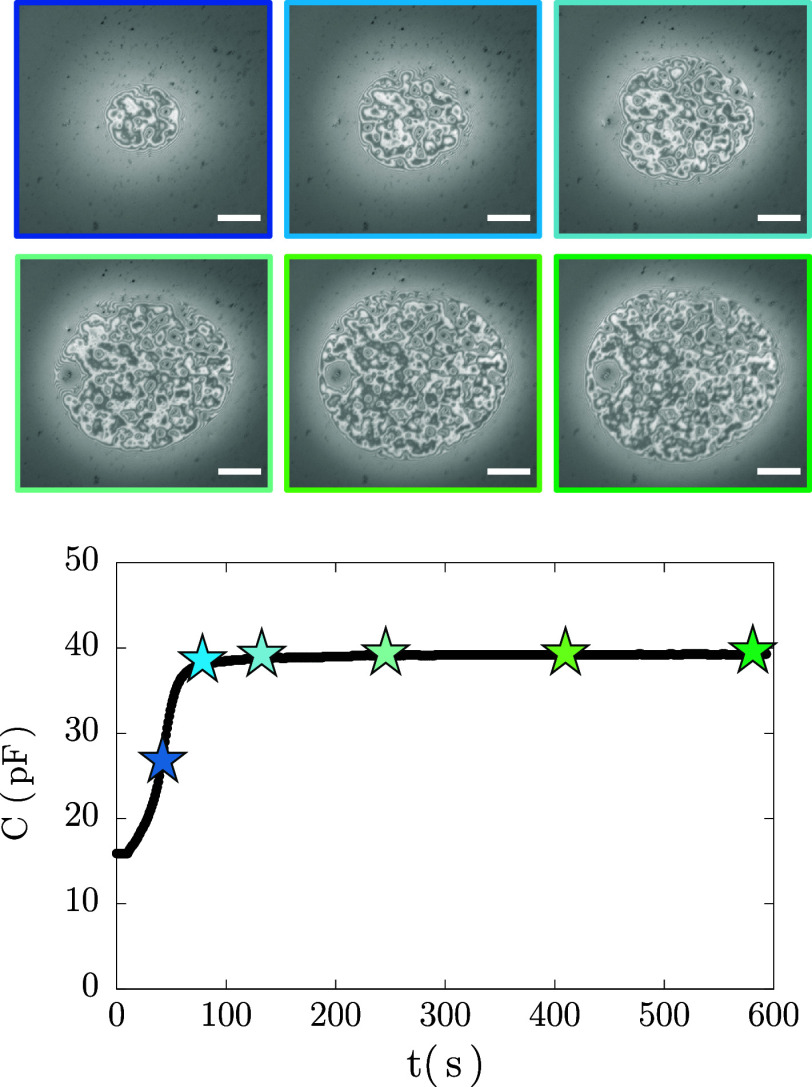
Drainage of asphaltene-laden film at Δ*P* = 50 Pa (*Ca* = 0.7): capacitance plot and representative images of drainage stages. The color of the image outlines corresponds to the stars in the plot. Scale bar: 100 μm.

Similarly to surfactant films, the thickness was estimated from the equilibrium capacitance, as shown in Figure 4. Notably, the film thicknesses are over an order of magnitude higher than those of the surfactant films, with *h*_eq_ ≈ 340 ± 18 nm. Given that the total film thickness is in the hundreds of nanometers, it is reasonable to assume that the influence of the asphaltene nanoaggregate layer on the overall dielectric constant of the film is minimal: from eq 20, a 10% deviation in the assumed dielectric constant would lead to an equivalent 10*%* error in the calculated film thickness, corresponding to an uncertainty of approximately ±30–40 nm for the asphaltene films. At the two lowest pressures (empty symbols), the calculated film thicknesses are smaller, likely due to the thin film area being comparatively small with respect to the total film area (i.e., *A*_f_ ≪ *A*_bikewheel_), making the thin film’s capacitance contribution negligible in the total measurement. It should be noted that creating films with controlled surface coverage is challenging due to the small volumes of spreading solution required, making quantitative agreement across measurements difficult. Figure S10b shows calculated thicknesses for films under the same Δ*P*, ranging from 180 to 280 nm. One experiment deviates significantly, likely due to higher surface coverage resulting in a thicker film.

The stability of thin asphaltene-covered films is remarkable. Asphaltene nanoaggregates, only a few nanometers in size, cannot explain the stability through steric interactions. Unlike surfactant films, which drain to thicknesses where intermolecular forces and disjoining pressure dominate – balancing van der Waals attraction or electric pressure against repulsive forces – these asphaltene films stabilize at much greater thicknesses, where intermolecular forces are negligible. Rather, the key mechanism lies in the mechanical strength of the asphaltene layers, which are known to exhibit an elastoplastic transition with a yield stress.^15^ When Δ*P* is applied, some liquid flows outward, exerting stress on the interface. If this stress is insufficient to deform the interface, drainage is arrested. The consistent thickness of stable films across different pressures highlights that stabilization is governed by the interface’s yield stress, emphasizing the critical role of interfacial rheological properties in complex flows.

Ideally, we aim to relate interfacial coverage in thin film balance (TFB) experiments to rheological properties. One approach is to subject the film to small amplitude oscillations, mimicking dilatational rheology, where surface stress and area deformations are used to calculate a dilatational modulus. This method requires the film behavior to be governed by interfacial properties, with interfacial layers compressing and expanding rather than the film thinning and reforming. We verified that film thickness remains constant during oscillations, as shown in Figure S12, where intensity profiles exhibit no significant changes over four oscillation cycles. The bulk film pressure was oscillated with an amplitude of ∼10 Pa at three different frequencies (0.02, 0.05, and 0.1 Hz). A surface stress amplitude can be estimated using ΔP = 2σ_s_/*R*, which yields σ_s_ ≈ 2.4–2.7 × 10^–3^ Pa m. Area changes are measured from image analysis, specifically the minimum (*A*_min_) and maximum (*A*_max_) areas during an oscillation cycle, to calculate the area deformation amplitude which is equal to α ≈ 2.5%. A dilatational modulus is then calculated as the ratio between the stress and strain amplitudes and yields K* ≈ 0.07–0.1 Pa m. The resulting dilatational modulus is shown in Figure 11 alongside interfacial dilatational rheology data from ref 15, demonstrating excellent agreement with dense asphaltene layers.

**Figure 11:**
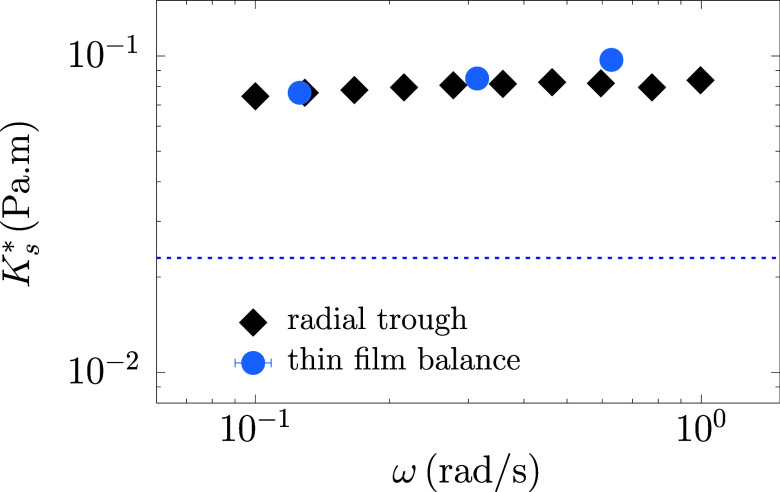
Dilatational frequency sweep for dense asphaltene layers: complex dilatational modulus K* vs frequency comparing data from radial trough experiments^15^ with area oscillations in the thin film balance.

In the thin film drainage problem the interface is mainly resisting being expanded, so that it is more insightful to estimate a dilatational yield stress σ_s,y_. Using the (easier to measure) shear yield stress τ_s,y_ ≈ 3.0 × 10^–4^ Pa m reported in^15^ and assuming that there is at least one order of magnitude difference between shear and dilatational stresses (due to the effect of compression redistributing the load across the structure^86,87^ ), then σ_s,y_ ≈ 3.0 × 10^–3^ Pa m. This value is higher than the surface stresses estimated in the TFB experiments, and thus the films remain stable even under higher pressures and interfacial stresses, suggesting that the dilatational yield stress is likely even higher.

Such a quantitative agreement between thin film drainage behavior and interfacial rheological properties is noteworthy and, to the best of our knowledge, not commonly observed in the field. In a series of papers, Tchoukov and co-workers^9,45,88^ used the TFB to study diluted bitumen films and attributed the stability of their 50 nm-thick films to the buildup of a 3D network in the film and the existence of a small yield stress of the thin film with magnitude 10^–2^ Pa. More recently, Bochner et al.^48^ studied asphaltene-in-toluene solutions, reporting coalescence times of minutes and attributed the long drainage to hydrophobic interactions between asphaltene tails. These studies involved asphaltene-soluble oil phases, introducing challenges such as (i) controlling interfacial concentrations, (ii) requiring “ageing” times for asphaltenes to populate the interface, and (iii) spontaneous emulsification, which complicates experiments (see Figure S1).

We have demonstrated the strength of these asphaltene-laden films in withstanding thin film drainage, and it is attributed to their interfacial rheological properties. We believe this highlights a meaningful and fundamental difference in how asphaltene-laden films can stabilize emulsions – either via bulk gelation as suggested in previous works or via interfacial mechanical resistance – depending on the mode of layer formation and the geometry of confinement. Next, we investigate whether electric fields can disrupt these films, as has been observed for the surfactant films.

#### Rupture

Voltage-step experiments on stable films postdrainage (III) reveal that, unlike surfactant films, applying ΔΦ of up to 500 mV does not lead to film rupture under any conditions. As shown in Figure 12: (a) no current increase is observed, (b) no area changes occur across different Δ*P*, and (c) images confirm no visible changes, contrasting with Figure 7.

**Figure 12:**
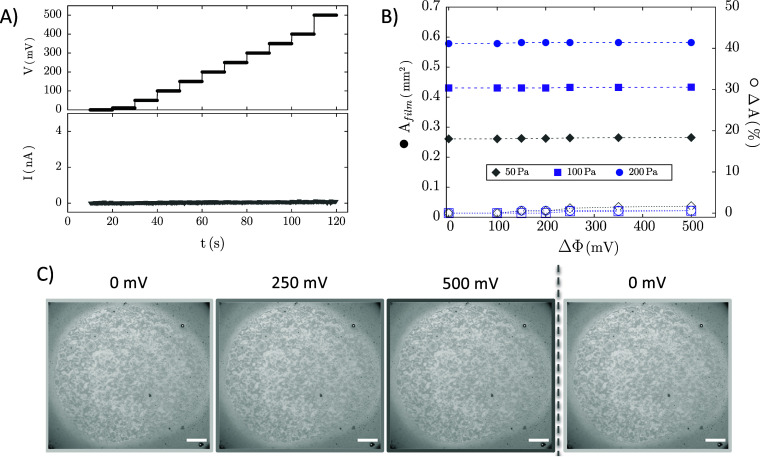
Typical voltage step experiments on stable asphaltene films: (a) applied potential and measured current; (b) Film area (filled symbols) and percentage area change (empty symbols) as a function of applied voltage steps. (c) Representative images of the experiment (scale bar = 100 μm). Gray data in (b) corresponds to the experiment depicted in (a) and (c).

This result can be explained by the electric pressure in the context of the pressure balance (eq 23). As shown in Figure 6 for a film thickness ∼300 nm, a ΔΦ = 400 mV generates an electric pressure of about 20 Pa. We have seen that for surfactant films even lower *P*_elec_ ∼ 10 Pa lead to film rupture as all contributions in the pressure balance were in the ∼10 Pa range. Here, additional mechanical stresses σ_*s*_ contribute, estimated as τ_s,y_/*h* ≈ 1000 Pa, thus strongly stabilizing the film against rupture. With our setup, the maximum electric pressure that could be achieved at such thicknesses is limited by the maximum voltage that can be applied and is equal to *P*_elect_ ≈ 400 Pa (or equivalently an electric field strength of 6.7 × 10^6^ V/m, see Figure S8b), which is insufficient to reach the ∼ kPa pressure range required to surpass the contributions due to mechanical stresses.

Thus, under the experimentally accessible conditions, asphaltene-laden interfaces could only be ruptured by introducing chemical demulsifiers, which are surface-active and can displace asphaltenes from the interface. Figure 13 shows experiments where demulsifier was added in situ through the aqueous phase after formation of a stable asphaltene film. Both films were drained at Δ*P* = 50 Pa and allowed to equilibrate for 30 min before starting a new capacitance experiment. At *t* = 60 s, demulsifier was added at concentrations of (a) 0.1 ppm and (b) 1 ppm. Results are plotted as dimensionless capacitance (*C*/*C*_0_) and area (*A*/*A*_0_), the experiment’s direct observables. While the area is seen to significantly increase, the ratio ϵ_f_ *h*_0_/*h* mildly decreases, but then stays constant throughout.

**Figure 13:**
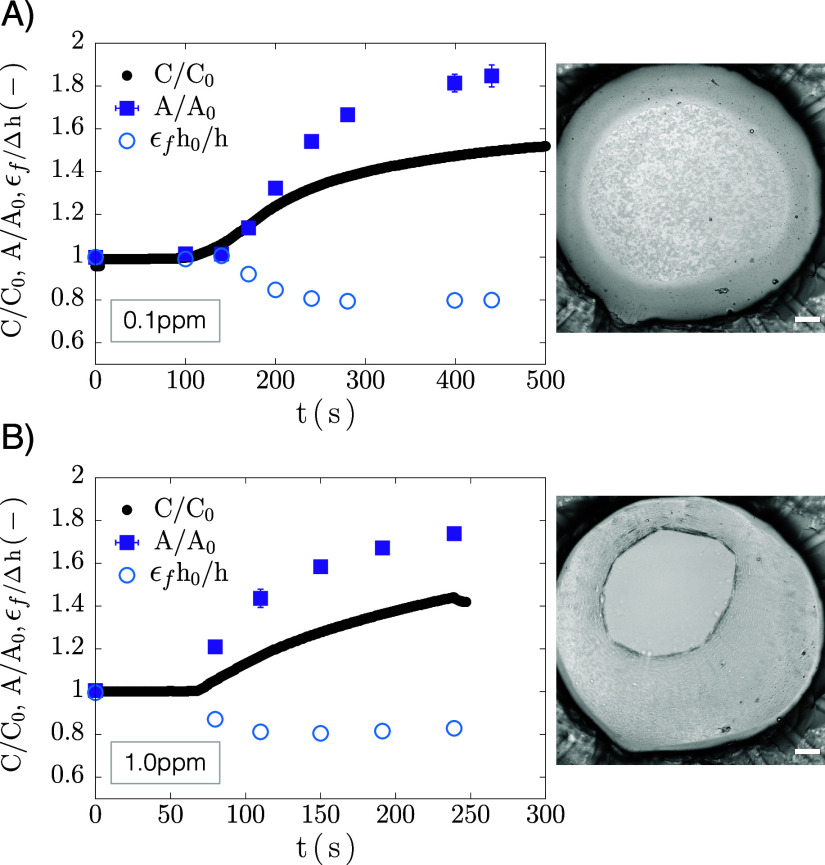
Demulsifier addition to asphaltene-stabilized films at a concentrations of (a) 0.1 ppm and (b) 1.0 ppm. Left: capacitance curves (black curve), normalized film area changes (purple data points), and normalized thickness (empty blue markers) as a function of experimental time. Introduction of demulsifier occurs at *t* = 60 s. Right: Representative images of each experiment. Scale bar: 100 μm.

In both cases, adding demulsifier causes the film to expand as the films drain further, indicated by increasing capacitance and film area. While the film in (a) expands but remains stable for at least 10 min, the film in (b) ruptures after a few minutes. Notably, rupture occurs as a puncture first forms at the interface, followed by propagation of a crack, as shown in the image sequence in Figure S13. This behavior highlights the solid-like nature of these films.

The effect of the demulsifier is also apparent in the interfacial shear rheology, as shown in Figure 14. Shortly after asphaltene spreading (brown arrow), a viscoelastic layer forms and the interfacial shear moduli stabilize shortly after with *G′* > *G* *″*. Upon demulsifier incorporation (red arrow), the moduli drop instantly, with a ∼ two-order-of-magnitude decrease observed for 50 and 5 ppm, and *G* *″* surpasses *G′* indicating a transition to more viscous behavior. Interestingly, for 0.5 ppm, the moduli decrease to a lesser extent and *G′* remains higher than *G* *″* indicating that a viscoelastic interface is preserved. A control-experiment of the pure demulsifier interface (Figure S11) shows simple viscous behavior, meaning that the remaining elastic nature of the interface in the 0.5 ppm curve must come from the asphaltenes. These results reveal that the extent of asphaltene displacement by demulsifier molecules depends on concentration, leading to either partial or complete removal. This highlights the value of interfacial shear rheology in understanding how demulsifiers weaken the elastic properties of interfacial layers. Moreover, the interfacial shear rheology results align qualitatively with the thin film balance experiments in Figure 13, hinting that local interfacial heterogeneities emerge upon demulsifier incorporation, leading to either (i) partial drainage with reduced elasticity but still preserving stability or (ii) film rupture when sufficient molecules adsorb. Due to the lack of more detailed information on the chemistry of the commercial demulsifier used, it is difficult to speculate further on the specific mechanisms that drive demulsifier incorporation into the asphaltene-laden interface.

**Figure 14:**
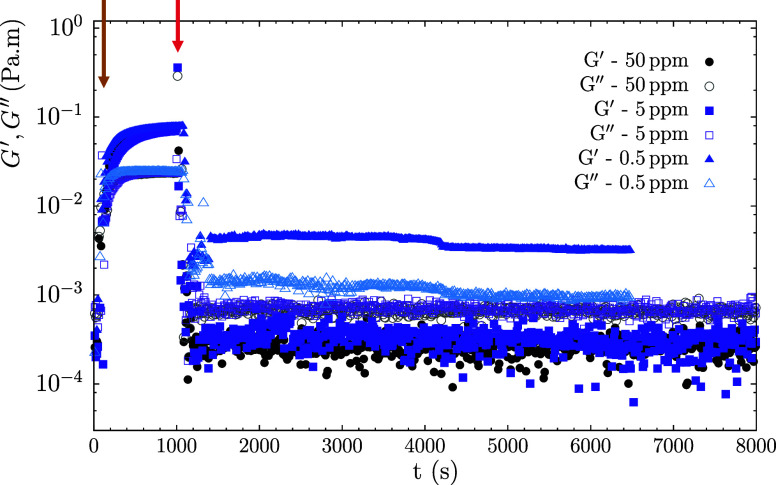
Oscillatory time sweeps (γ = 0.02% and ω = 2 rad/s) showing the evolution of the interface when demulsifier is added at different concentrations, namely 0.5 ppm (blue curves), 5 ppm (purple curves), and 50 ppm (black curves). Brown arrow indicates the moment of asphaltene spreading and red arrow indicates the moment of demulsifier incorporation.

Next, we investigate the effect of electric fields on films where demulsifier has adsorbed but still remained stable, such as the one depicted in Figure 13a. Applying voltage steps to these mixed films, unlike for pure asphaltene films, induces rupture at a certain voltage. Figure S14 shows the applied voltage steps and measured current and Movie S2 the full experiment. Representative images in Figure 15 capture the interface at (0 mV), the last frame before rupture at 400 mV, and the first frame after rupture, clearly showing that the film fractures at a critical voltage, consistent with the solid state nature of the interface.

**Figure 15:**
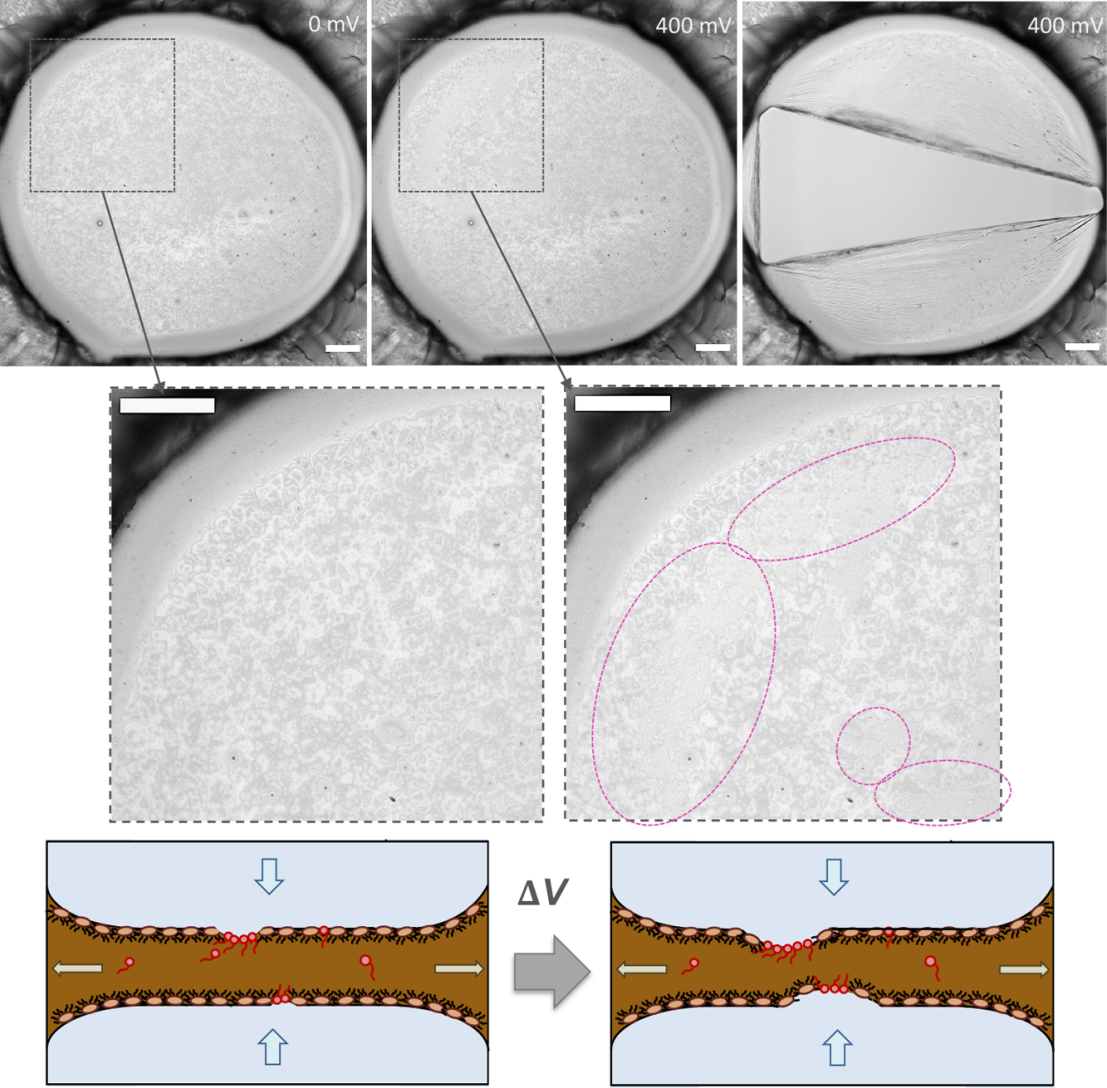
Breakup of mixed asphaltene+demulsifier film under stepwise increase in ΔΦ: images of the film: initial film at 0 mV, at 400 mV (last frame before rupture) and the first frame upon rupture - bottom row: zoomed in view. Scale bar: 100 μm – Schematics of the proposed mechanism for synergestic action of demulsifiers and electric fields in water-in-crude oil emulsions.

To understand this behavior, we examine the film images in Figure 15, particularly the two insets from the dashed square region. As the voltage increases, specific areas of the film (highlighted by pink dashed regions) become denser and greyer, indicating a local decrease in thickness. These regions likely correspond to areas of demulsifier adsorption, creating a heterogeneous mixed layer as seen by comparing pre- and postrupture frames. The local thickness reduction, once again, amplifies the electric field strength until a critical voltage or pressure is reached, causing rupture. Interestingly, the current data (Figure S14) shows an increase before rupture (*t* ∼ 90 s), hinting at pore formation, similar to surfactant layers. This aligns with Skartlien et al.,^57^ who suggested demulsifiers weaken regions of lower line tension, enabling electroporation under similar field strengths (1–18 kV/cm). Here, we attribute this primarily to local thickness reduction, as electroporation occurs in very thin regions (∼ few nm).

This mechanism, shown schematically in Figure 15 illustrates how asphaltene-stabilized films break. Thick films are only mildly affected by electric fields due to low electric pressure. However, when demulsifiers adsorb and locally thin the film, the electric field is locally amplified, causing further thinning and eventual rupture. Electric fields alone are often inefficient in breaking crude oil/asphaltene-stabilized emulsions and these results highlight the synergistic efficiency of combining demulsifiers and electric fields, as demulsifiers help localize the electric field. Moreover, our results shed light on why tiny amounts of demulsifiers (at ppm concentrations) suffice to enhance coalescence. While Tchoukov et al.^45^ suggest using demulsifiers to break the bulk yield stress, with our findings we propose designing demulsifiers that effectively penetrate interfacial layers, offering a promising approach to reduce the ecological impact of these chemicals.

## Conclusions

We demonstrated that local thickness variations and the subsequent enhancement of the electric field (and the local Maxwell stress) play a crucial role in promoting electro-coalescence in emulsion films, both for simple (surfactant-based) and complex (asphaltene-laden) interfaces. By carrying out experiments in a electro-dynamic thin film balance, we showed how this difference in surface activity (interfacial tension vs interfacial rheology) remarkably affects drainage and rupture.

Nonionic surfactant films drain and form thin Newton black films (with thicknesses ∼10 nm), stabilized by intermolecular forces but susceptible to rupture under electric pressures in the ∼ kPa range. When an electric field is applied during drainage, lower electric pressures (∼ Pa) are sufficient to destabilize the thicker films, suggesting more efficient pathways to promote electrocoalescence. We interpret these results through pressure balance considerations at different drainage stages. Combined with FEM simulations, our analysis shows that the locally very small film thickness amplifies the local electric field, facilitating rupture at low voltages.

In contrast, asphaltene-laden interfaces are elastoplastic and also form stable, but much thicker films (∼350 nm), making electric fields less effective. While in earlier works this “3D network” forms a gel of high viscosity, thus a bulk effect, for the asphaltene interfaces used here we propose that the interfacial yield stress arrests the drainage of the thin liquid Newtonian film of hexadecane, as the interface serves as a boundary condition for the thin film flow. In this way, our results can also be generalized to other complex interfaces, such as e.g. particle-stabilized interfaces that also possess an interfacial yield stress. Only when demulsifiers are added, even at very small amounts partially displacing asphaltenes at the oil–water interface and creating local thin spots, can the electric field be enhanced enough to cause film rupture.

These findings show that controlling local film thickness – and thereby amplifying the electric field at targeted regions – is key to efficient emulsion destabilization. This perspective of focusing on how to handle dense interfacial layers in electrocoalescence is important not only for crude oils with increased degree of complexity but also for other stabilizers that impart pronounced mechanical properties to the liquid interface, and could inform better strategies for destabilizing such interfaces, particularly regarding the interplay of electric fields and demulsifiers.

## Supplementary Material







## Notes

Extra information on creation of the insoluble asphaltene interfaces; TFEM simulation details; and supporting results for drainage of surfactant and asphaltene-laden films (PDF)
Movie corresponding to the experiment shown in Figures 7 and 8 in the main text: rupture of a stable surfactant thin film under increasing voltage steps (AVI)
Movie corresponding to the experiment shown in Figure 15 in the main text and Figure S14: rupture of a mixed asphaltene + demulsifier thin film under increasing voltage steps (AVI)
