# Supplementary material for: Mechanistic Insights into Emulsion Destabilization by Electric Fields
Source: Langmuir. 2025 Sep 22;41(39):26589–604. doi: 10.1021/acs.langmuir.5c02307 (PMC12509331; doi:10.1021/acs.langmuir.5c02307)
Supplement: Supplementary file 1 [file la5c02307_si_001.pdf]

Supporting Information: Mechanistic Insights into Emulsion Destabilization by Electric Fields

Alexandra Alicke,*,†,‡ Nick O. Jaensson,*,‡ and Jan Vermant*,†

†*Department of Materials, ETH Zurich, Vladimir-Prelog-Weg 5, Zurich 8093, Switzerland*

‡*Department of Mechanical Engineering, Eindhoven University of Technology, P.O. Box 513, 5600 MB Eindhoven, The Netherlands*

E-mail: a.alicke@tue.nl; n.o.jaensson@tue.nl; jan.vermant@mat.ethz.ch

Supporting Movies

Movie S1: Movie corresponding to the experiment shown in Figures 7 and 8 in the main text: rupture of a stable surfactant thin film under increasing voltage steps (real time).

Movie S2: Movie corresponding to the experiment shown in Figure 15 in the main text and Figure S14: rupture of a mixed asphaltene + demulsifier thin film under increasing voltage steps (fast forwarded 2x).

Materials and Methods

Creating asphaltene-laden interfaces

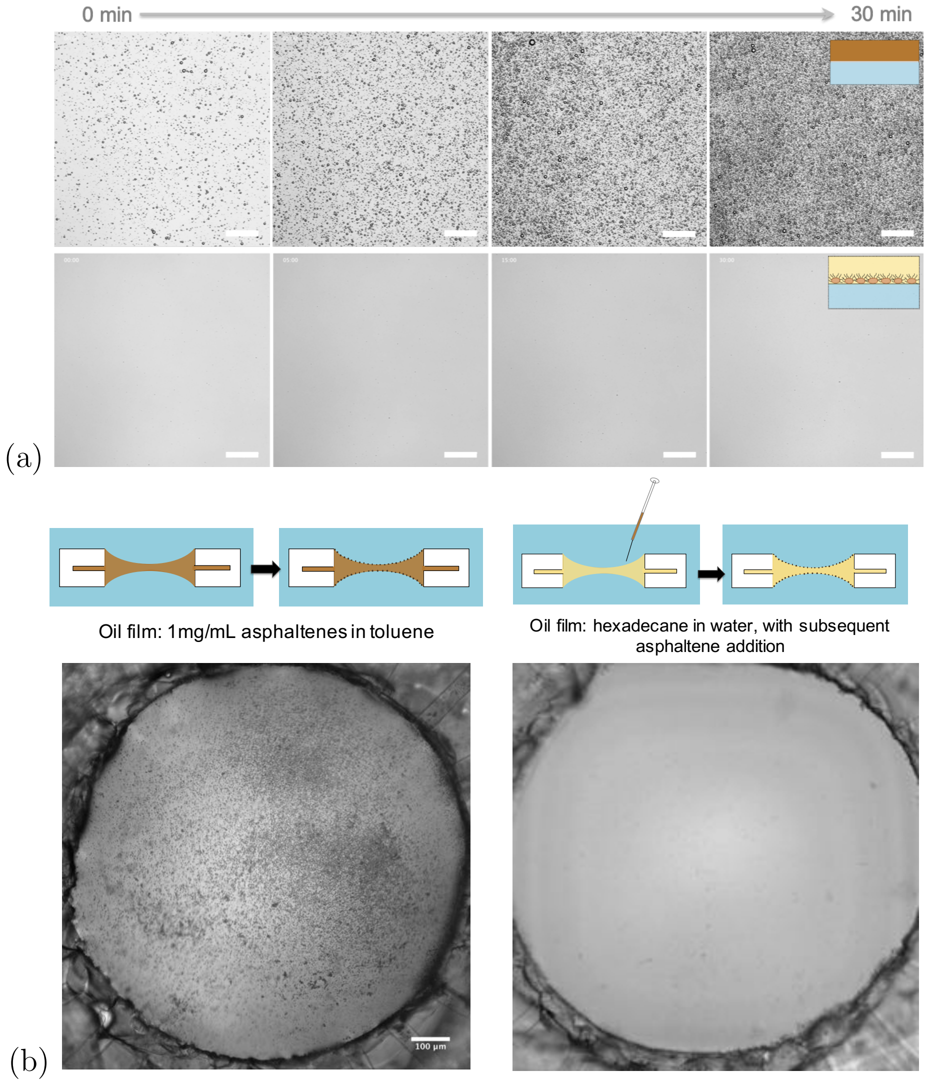

Figure S1: Different methods of creating asphaltene films: by adsorption from an oil film composed of a good solvent (asphaltene-in-toluene solution), and by using the spreading method detailed in the main text, namely of creating insoluble layers by direct spreading onto the liquid-liquid interface. (a) sample experiments on a flat interface: different columns correspond to experimental times of 0, 5, 15, and 30 minutes. Scale bars correspond to 200μm. (b) in thin film drainage experiments. Spontaneous emulsification clearly occurs in the case of the asphaltene-soluble films within a timeframe of 30 minutes.

Detailed pressure balance contributions

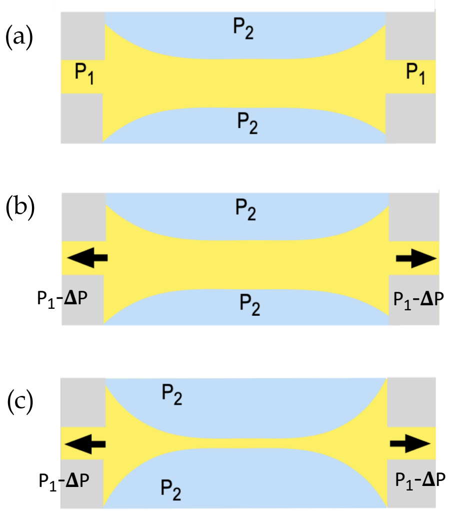

$$P_2 + P_L = P_1$$

$$P_{\mathrm{E}} + P_2 + P_L = P_1 - \Delta P$$

$$P_{\mathrm{E}} + P_2 + P_L = P_1 - \Delta P + P_H(h,r) + \Pi_{vdW}(h,r) + \Pi_{steric}(h,r) + \frac{\sigma}{2r}\frac{\partial}{\partial r}\left(r\frac{\partial h}{\partial r}\right)$$

Figure S2: Illustration of the pressure contributions in the film at different stages (not drawn to scale): (a) mechanical equilibrium in the thick film; (b) when a pressure drop ΔP is applied to the film; (c) the thin film in equilibrium. The different pressure contributions are explained in the text. Illustration adapted from[S1].

FEM simulation details

We provide here the full details of the finite-elements numerical simulations of electrocoalescence in the thin-film balance of which we reported the salient results in the main manuscript.

We consider the domain depicted in Fig. S3, which is an axisymmetric representation of the thin-film balance. The film and drop domain are denoted by Ω_1 and Ω_2 respectively, whereas the interface between them is denoted by Γ_{i}. The normal to the interface is denoted by $\boldsymbol{n}$ and assumed to point from Ω_1 to Ω_2, as shown in the figure. The boundaries Γ_{w} and Γ_{out} are the rigid walls and outflow boundary, respectively. Moreover, Γ_{s} describes the surface in the r-θ plane through the center of the film, and symmetry (for the flow) and anti-symmetry (for the electric potential) is assumed in Γ_{s}.

As explained in Section 2 of the main manuscript, the momentum balance and mass

balance reduce to

$$-\nabla \cdot (\boldsymbol{\sigma}_{\text{mech}} + \boldsymbol{\tau}_{\text{M}}) = \mathbf{0} \qquad \text{in } \Omega_1 \cup \Omega_2, \tag{1}$$

$$\nabla \cdot \boldsymbol{u} = 0 \qquad \text{in } \Omega_1 \cup \Omega_2, \tag{2}$$

where $\boldsymbol{u}$ is the fluid velocity and $\boldsymbol{\sigma}_{\text{mech}}$ is the hydrodynamic Cauchy stress tensor given by

$$\boldsymbol{\sigma}_{\text{mech}} = -p\boldsymbol{I} + 2\eta\boldsymbol{D}, \tag{3}$$

where η is the viscosity (assumed equal for both domains), $\boldsymbol{D} = \left(\nabla\boldsymbol{u} + (\nabla\boldsymbol{u})^T\right)/2$ is the rate-of-strain tensor, p is the pressure and $\boldsymbol{I}$ is the unit tensor.

Furthermore, $\boldsymbol{\tau}_{\text{M}}$ is the Maxwell stress tensor given by

$$\boldsymbol{\tau}_{\text{M}} = \epsilon\left(\boldsymbol{E}\boldsymbol{E} - E^2\boldsymbol{I}/2\right), \tag{4}$$

where ϵ is the permittivity, $\boldsymbol{E}$ is the electric field vector and $E = \sqrt{\boldsymbol{E}\cdot\boldsymbol{E}}$ is the magnitude of the electric field vector. Note, that the Maxwell stress tensor is zero in the conducting medium and isotropic in the dielectric medium. We only take the effect at Γ_{i} into account by absorbing the isotropic term in the pressure[S2].

It is assumed that Ω_1 is a perfect dielectric medium, whereas Ω_2 is a perfect conducting medium. The electric potential field, denoted by ϕ, is only relevant in Ω_1 and satisfies

$$\nabla^2\phi = 0 \qquad \text{in } \Omega_1. \tag{5}$$

The electric field can be obtained from the potential field via

$$\boldsymbol{E} = -\nabla\phi. \tag{6}$$

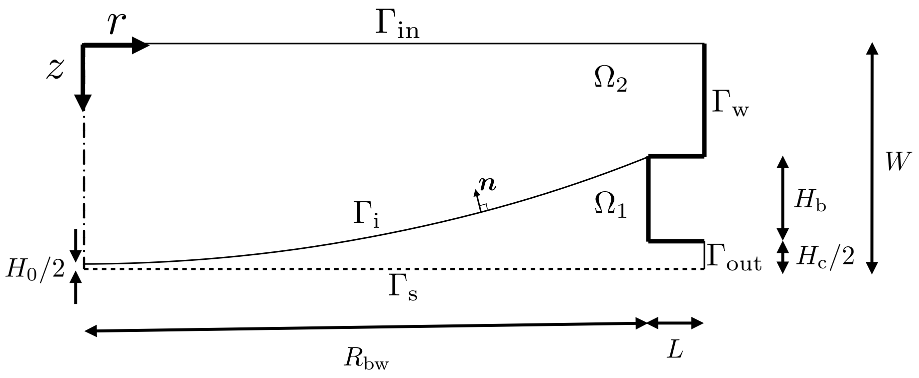

Figure S3: Schematics of the drainage problem and domain for FEM simulations, indicating the coordinate system used, relevant dimensions and boundary conditions.

The movement of the interface is tracked in a Lagrangian manner and is thus described by

$$\frac{d\boldsymbol{x}_\mathrm{s}}{dt} = \boldsymbol{u}, \tag{7}$$

where $\boldsymbol{x}_\mathrm{s}$ are the coordinates of the interface.

Initial and boundary conditions

In order to solve Eq. (7), the initial location of the interface is needed. We assume that the shape of the interface is initially part of a spherical surface, with a radius and location fixed by the corner point of the solid boundary and the location of the interface at the center line, which is determined by H_0 (see Fig. S3). The initial radius of curvature of the interface is denoted by R_0. At the interface, we assume a no slip condition for the velocity:

$$[\![\boldsymbol{u}]\!] = \boldsymbol{0}, \tag{8}$$

where $[\![\,]\!]$ indicates a jump across the interface ('fluid 1 − fluid 2'). Furthermore, we assume the existence of a constant interfacial tension, as well as mechanical extra stresses, resulting

in the following stress balance[S2–S4]:

$$[\![\boldsymbol{\sigma}_{\text{mech}} \cdot \boldsymbol{n}]\!] + [\![\boldsymbol{\tau}_{\text{M}} \cdot \boldsymbol{n}]\!] = -\sigma(\nabla_{\text{s}} \cdot \boldsymbol{n})\boldsymbol{n} + \nabla_{\text{s}} \cdot \boldsymbol{\tau}_{\text{s}} \tag{9}$$

where $\boldsymbol{\tau}_{\text{s}}$ is the interfacial extra stress tensor and $\nabla_{\text{s}} = \boldsymbol{I}_{\text{s}} \cdot \nabla$ is the surface gradient operator with $\boldsymbol{I}_{\text{s}} = \boldsymbol{I} - \boldsymbol{n}\boldsymbol{n}$ the surface unit tensor. Note, that since $\boldsymbol{\tau}_{\text{M}} = \boldsymbol{0}$ in Ω_2, the jump in Maxwell stress reduces to $[\![\boldsymbol{\tau}_{\text{M}} \cdot \boldsymbol{n}]\!] = \boldsymbol{\tau}_{\text{M}} \cdot \boldsymbol{n}$, where $\boldsymbol{\tau}_{\text{M}}$ is the Maxwell stress tensor evaluated at the Ω_1-side of the interface. In this work we assume that there exists a shear viscosity at the interface[S5,S6]:

$$\boldsymbol{\tau}_{\text{s}} = 2\eta_{\text{s}}(\boldsymbol{D}_{\text{s}} - \text{tr}(\boldsymbol{D}_{\text{s}})\boldsymbol{I}_{\text{s}}/2), \tag{10}$$

where η_{s} is the interfacial shear viscosity and $\boldsymbol{D}_{\text{s}}$ is the surface rate-of-deformation tensor given by

$$\boldsymbol{D}_{\text{s}} = (\nabla_{\text{s}}\boldsymbol{u} \cdot \boldsymbol{I}_{\text{s}} + \boldsymbol{I}_{\text{s}} \cdot (\nabla_{\text{s}}\boldsymbol{u})^T)/2. \tag{11}$$

For the flow problem, symmetry conditions are applied at Γ_{s} and axisymmetry conditions are applied at $r = 0$. Furthermore, we assume no-slip at the solid walls, and an imposed pressure P_{out} at the outlet, which results in:

$$\boldsymbol{u} = \boldsymbol{0} \qquad \text{on } \Gamma_{\text{w}}, \tag{12}$$

$$\boldsymbol{\sigma}_{\text{mech}} \cdot \boldsymbol{n} = P_{\text{out}}\boldsymbol{n} \qquad \text{on } \Gamma_{\text{out}}, \tag{13}$$

$$\boldsymbol{\sigma}_{\text{mech}} \cdot \boldsymbol{n} = \boldsymbol{0} \qquad \text{on } \Gamma_{\text{in}}. \tag{14}$$

The imposed pressure at the outlet is split into two parts:

$$P_{\text{out}} = 2\sigma/R_0 + \Delta P, \tag{15}$$

where the first term on the right hand side is the equilibrium (Laplace) pressure, which ensures that no flow occurs in the absence of an electric field, and ΔP is the drainage pressure, i.e., the pressure in addition to the equilibrium pressure.

For the electric potential, only boundary conditions for the film domain (Ω_1) are required. We assume that the potential at the interface Γ_i is equal to the imposed potential and that the solid walls are insulating. Furthermore, an anti-symmetry condition is imposed on Γ_s, which yields:

$$\phi = \phi_\mathrm{w}/2 \qquad \text{on } \Gamma_\mathrm{i}, \tag{16}$$

$$\boldsymbol{n} \cdot \nabla\phi = 0 \qquad \text{on } \Gamma_\mathrm{w}, \tag{17}$$

$$\phi = 0 \qquad \text{on } \Gamma_\mathrm{s}, \tag{18}$$

where ϕ_w is the imposed potential across the full film thickness. Note that due to the symmetry conditions imposed here, the potential will range from ϕ_w at the top of the film to $-\phi_\mathrm{w}$ at the bottom of the film.

Numerical method

The momentum and mass balance (Eqs. (1) and (2)) and the Laplace equation for the potential (Eq. (9)) form a set of coupled equations which are solved using the finite element method. We use iso-parametric, triangular P2/P1 (Taylor-Hood) elements for the velocity/pressure fields, as well as P2 elements for the potential field. To integrate the system in time, first within a time step a second-order prediction is made for the location of the interface, after which the nodes of the mesh are updated accordingly. Then Eq. (9) is solved to find ϕ, from which the $\boldsymbol{E}$ field is obtained using Eq. (6). With $\boldsymbol{E}$, the Maxwell stress tensor is calculated and used to solve Eqs. (1) and (2), together with the interfacial stress balance Eq. (9) for the velocity $\boldsymbol{u}$ and pressure p. Note that the two domains are meshed sep-

arately and Lagrange multipliers are used to connect the velocity while allowing for a jump in pressure. Finally, the interface location is corrected by solving Eq. (7) using a second-order time-integration scheme. Gmsh[S7] is used for generating the meshes and remeshing is performed when the elements become too distorted. More information about the numerical method can be found in previously published work[S8–S10].

The parameters used in the simulations are summarized in Table S1.

Table S1: Parameters used in the simulations.

Parameter	Value
W	0.4
$H_0/2$	0.01
$H_c/2$	0.05
H_b	0.15
R_{bw}	1
L	0.1
η	1
η_s	0 or 1000
σ	10
$\phi_w/2$	2
ϵ	2×10^{-4}
ΔP	32.7

Mesh-convergence

To study mesh-convergence, we consider three initial meshes of increasing resolution. Note, that due to remeshing, these meshes change during the simulation, but their characteristics remain the same. The meshes, named M1, M2 and M3, differ by the amount of nodes at the interface (N_i), and are summarized in Table S2. An example of mesh M3 is shown in Fig. S4.

In Fig. S5 we present the thickness of the interface versus time as computed on the different meshes. For these simulations, a time-step size of $\Delta t = 0.01$ is used. The results indicate that all meshes give very similar results, with some minor deviations on M1 visible

Table S2: Meshes used in the mesh-convergence study.

Mesh	N_{i}	number of nodes
M1	20	979
M2	40	2155
M3	80	4159

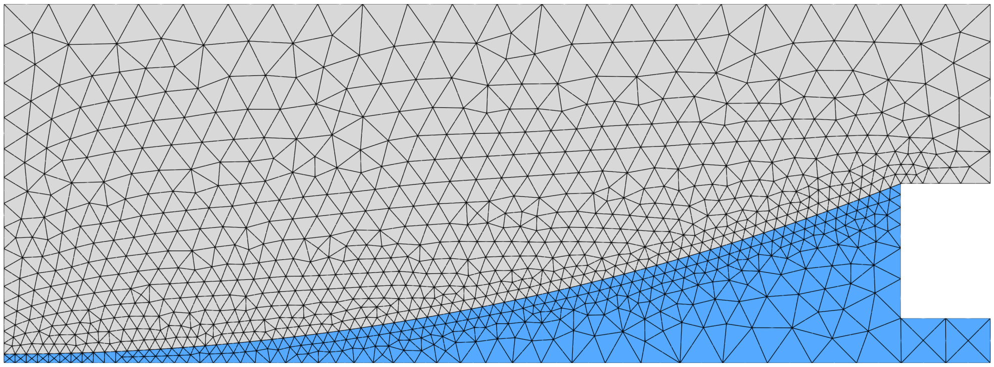

Figure S4: Mesh M3.

towards the end of the simulation. Moreover, on M1 the simulations fail much earlier as compared to M2 and M3, due to the film becoming very thin. For mesh M2 and M3, the results are almost indistinguishable.

Time-convergence

In Fig. S5 we present the thickness of the interface versus time as computed for time step sizes of $\Delta t = 0.01$, 0.02 and 0.04. These simulations are performed using M2. For the stress-carrying interface, the results between the different time-step sizes are indistinguishable. For the interface with $\eta_{\mathrm{s}} = 0$, where the dynamics are much faster, there are some deviations visible towards the end of the simulations.

The simulations for the main manuscript (Figure 9) are performed on mesh M3 using a time step size of $\Delta t = 0.01$.

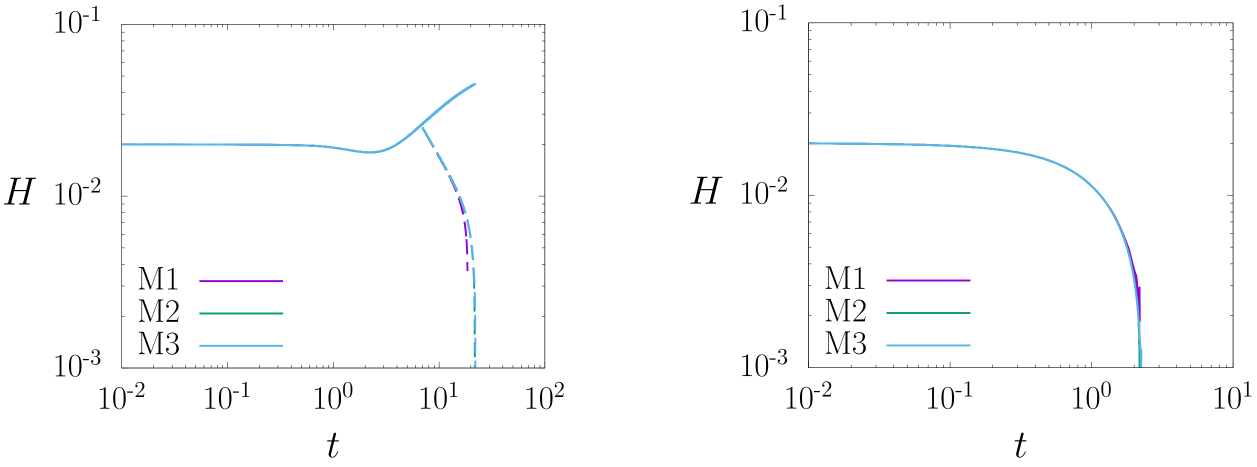

Figure S5: Mesh-convergence of the evolution of the center thickness (solid lines) and minimal thickness of the thin film (dashed lines). Left: $\eta_s = 1000$ and right: $\eta_s = 0$. Note that for $\eta_s = 0$, the film does not develop a dimple thus is the center thickness equal to minimal thickness.

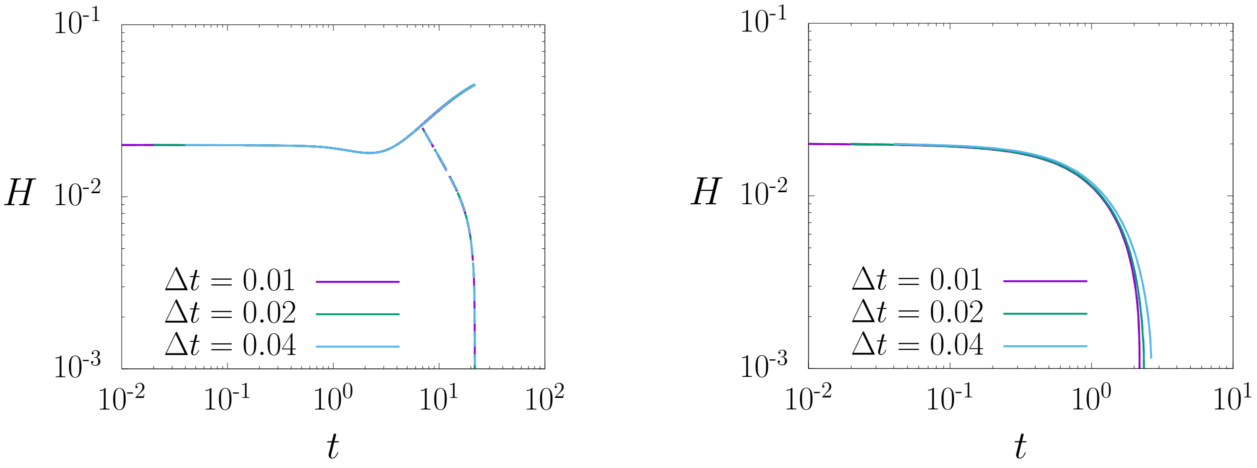

Figure S6: Time-convergence of the evolution of the center thickness (solid lines) and minimal thickness of the thin film (dashed lines). Left: $\eta_s = 1000$ and right: $\eta_s = 0$. Note that for $\eta_s = 0$, the film does not develop a dimple thus is the center thickness equal to minimal thickness.

Results

Thin film drainage: surfactants

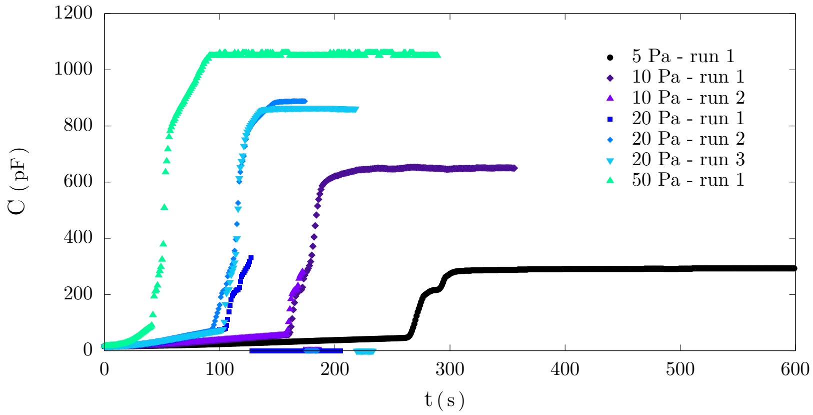

Figure S7: Span 80 films drained at different pressures: capacitance curves as a function of time for different applied pressures, sample data of multiple runs.

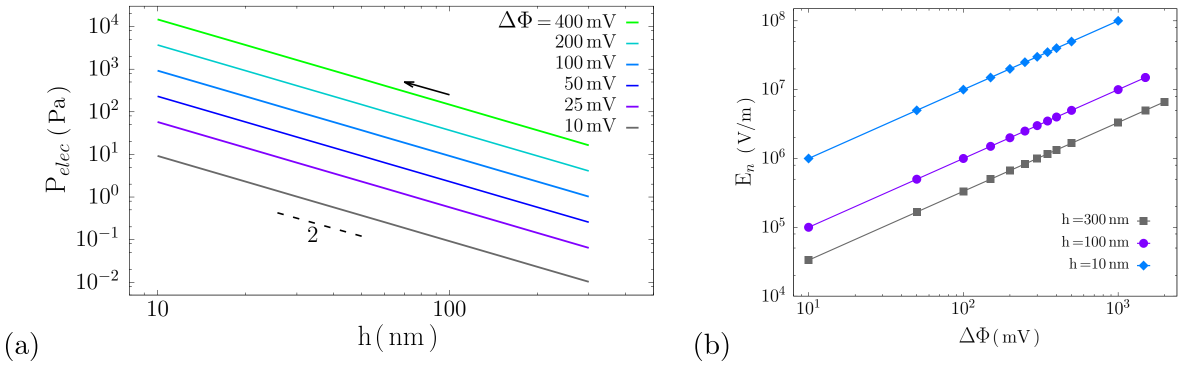

Figure S8: Electric pressure as a function of film thickness for different applied $\Delta\Phi$ (log x log scale) calculated using Eq. 9 in the main text. The arrow indicates the drainage direction. (b) Electric field strength as a function of $\Delta\Phi$ calculated for different thicknesses using Eq. 4 in the main text.

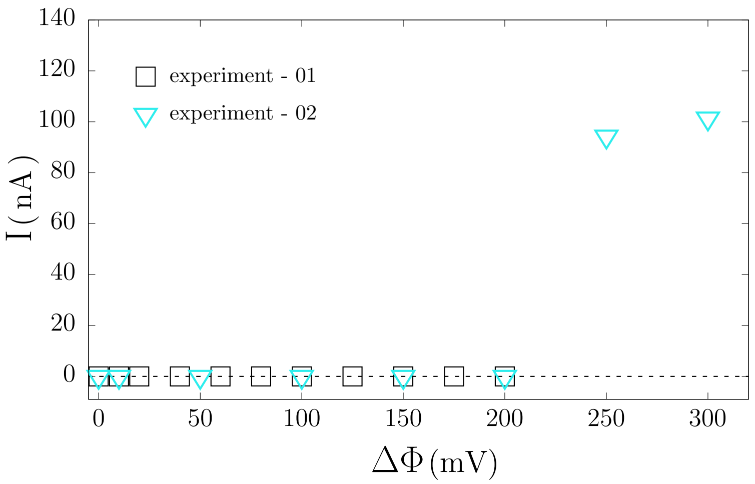

(a)

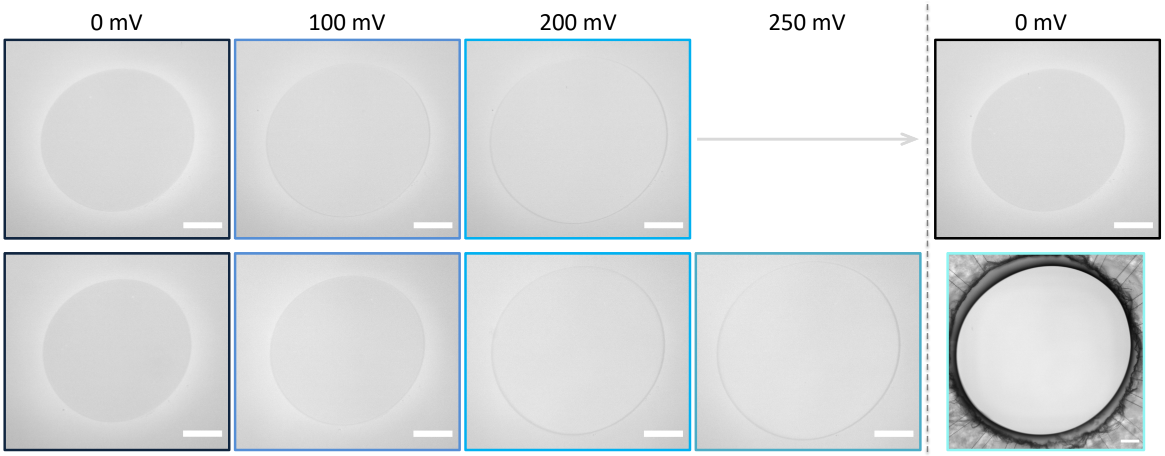

(b)

Figure S9: Voltage step experiments on a surfactant film: (a) measured current and (b) representative images as a function of applied potential for two experiments: experiment 1 ((a) □, (b) top row) and experiment 2 ((a) $\bigtriangledown$, (b) bottom row). In the first experiment the applied potential was increased to 200mV and the film did not break. However, in a subsequent step the same film was subjected to higher $\Delta\Phi$ and broke at 250 mV. Scale bar in all images equal to 100μm.

Thin film drainage: asphaltenes

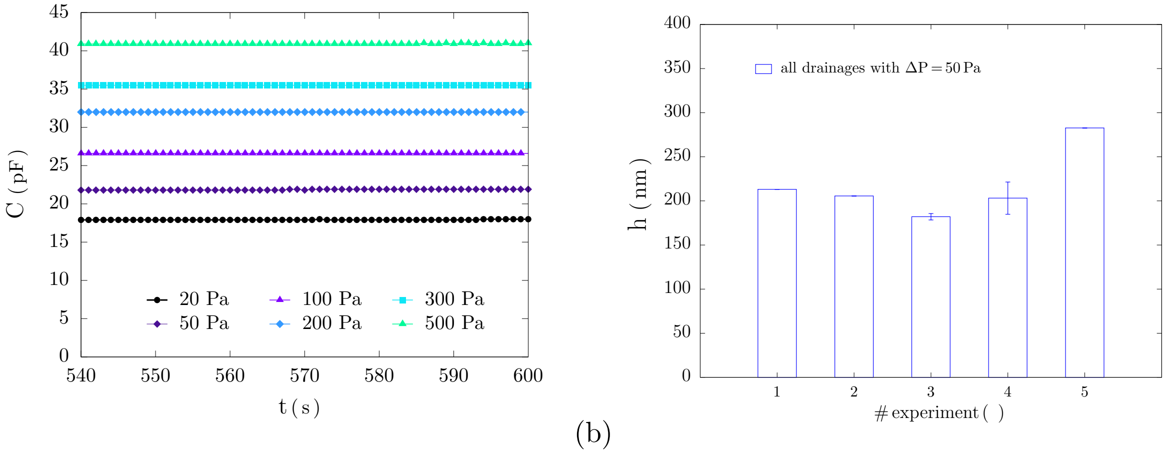

(a) (b)

Figure S10: (a) Equilibrium capacitance: last 60s of measured capacitance for different pressures; (b)Calculated thickness from capacitance for films drained at $\Delta P = 50\,\text{Pa}$ in different experiments (i.e. different created films. Errorbars correspond to differences in thickness between drainage steps for the same film.

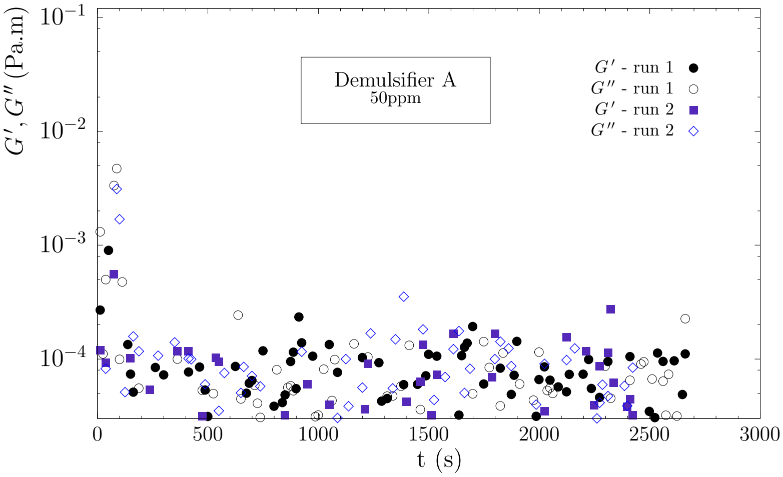

Figure S11: Interfacial shear rheology experiments: control experiment of pure demulsifier interface, showing negligible moduli and indicating purely viscous behavior as expected typical for a surfactant.

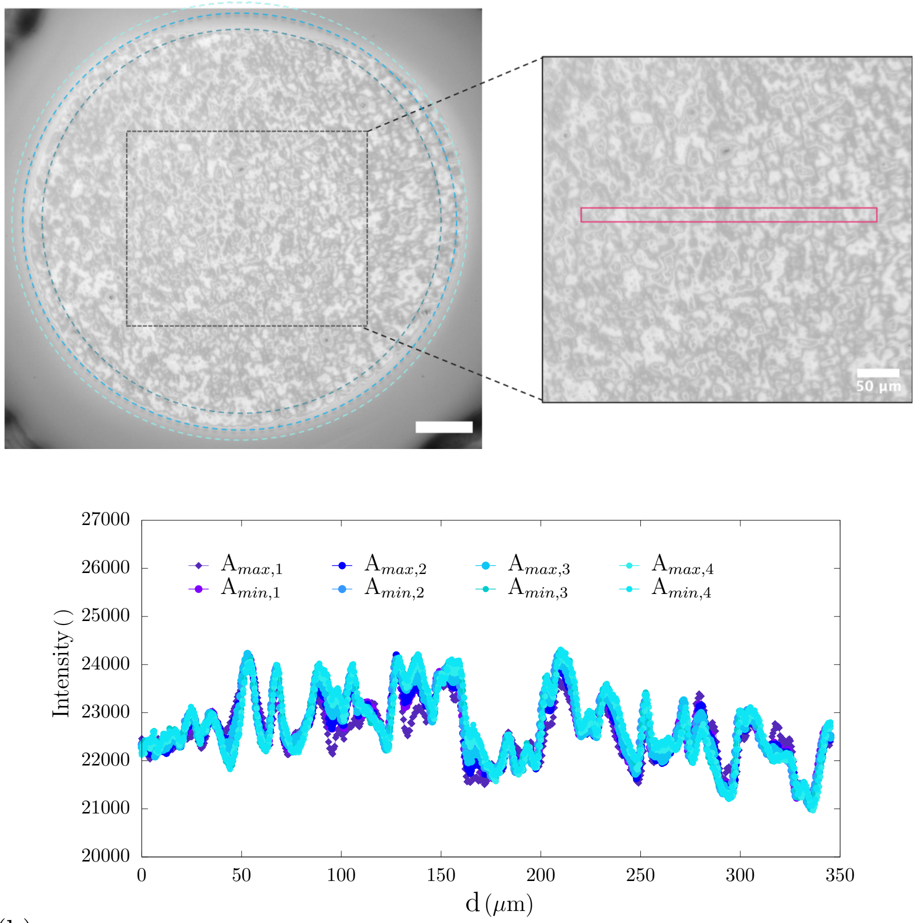

(a)

(b)

Figure S12: Oscillatory area sweep measurements in the thin film balance: An asphaltene-laden film that was drained at 100Pa to an equilibrium thickness of ∼240nm is oscillated with a (bulk) pressure amplitude of 10Pa and the area changes are quantified with image analysis (a) specifically the minimum and maximum areas, A_{min} and A_{max}, at maximum compression and maximum dilation, respectively, as indicated by the dashed circles; (b) Intensity profiles measured in the center of the asphaltene films during four oscillations cycles, averaged in the area indicated by the red rectangle - no significant changes in intensity are observed, indicating that no changes in film thickness are occuring.

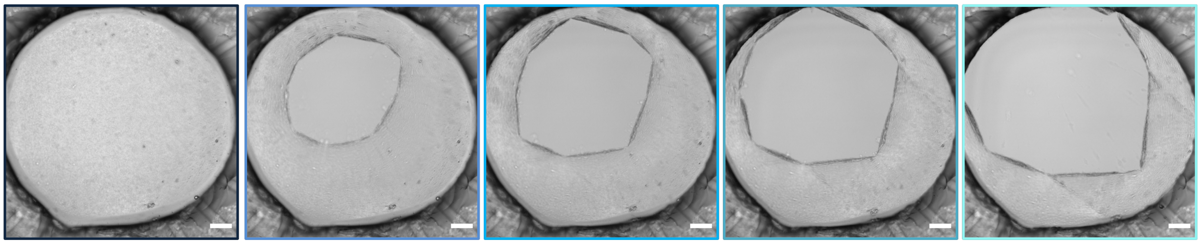

Figure S13: Sequence of images depicting the rupture of film in experiment reported in Fig.13(b) in the main text.

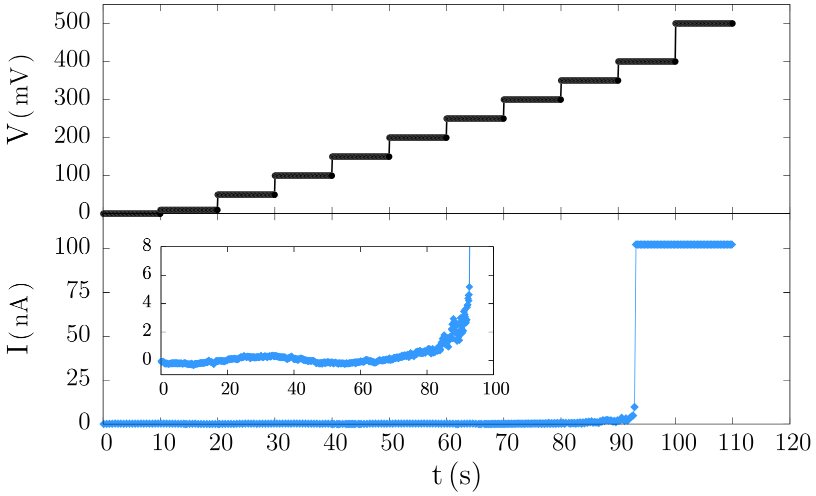

Figure S14: Voltage step experiment carried on mixed films of asphaltene and demulsifier: applied potential and measured current, corresponding to images in Fig.14 in the main text.

References

(S1) Chatzigiannakis, E.; Vermant, J. Breakup of Thin Liquid Films: From Stochastic to Deterministic. *Phys. Rev. Lett.* **2020**, *125*, 158001.

(S2) Saville, D. A. Electroydrodynamics: The Taylor-Melcher Leaky Dielectric Model. *Annu. Rev. Fluid Mech.* **1997**, *29*, 27–64.

(S3) Baygents, J. C.; Rivette, N. J.; Stone, H. A. Electrohydrodynamic deformation and interactions of drop pairs. *J. Fluid Mech.* **1998**, *368*, 359–375.

(S4) Jaensson, N. O.; Anderson, P. D.; Vermant, J. Computational interfacial rheology. *J. Non-Newtonian Fluid Mech.* **2021**, *290*, 104507.

(S5) Boussinesq, M. J. Sur l'existence d'une viscositéseperficielle, dans la mince couche de transition séparant un liquide d'un autre fluide contigu. *Ann. Chim. Phys.* **1913**, *29*, 349.

(S6) Scriven, L. Dynamics of a fluid interface Equation of motion for Newtonian surface fluids. *Chem. Eng. Sci.* **1960**, *12*, 98–108.

(S7) Geuzaine, C.; Remacle, J.-F. Gmsh: A 3-D finite element mesh generator with built-in pre- and post-processing facilities. *Int. J. Numer. Methods Eng.* **2009**, *79*, 1309–1331.

(S8) Balemans, C.; Hulsen, M. A.; Anderson, P. D. Modeling of complex interfaces for pendant drop experiments. *Rheol. Acta* **2016**, *55*, 801–822.

(S9) Jaensson, N.; Hulsen, M.; Anderson, P. On the use of a diffuse-interface model for the simulation of rigid particles in two-phase Newtonian and viscoelastic fluids. *Computers & Fluids* **2017**, *156*, 81–96, Ninth International Conference on Computational Fluid Dynamics (ICCFD9).

(S10) Carrozza, M.; Hulsen, M.; Anderson, P. Benchmark solutions for flows with rheologically complex interfaces. *J. Non-Newtonian Fluid Mech.* **2020**, *286*, 104436.
